# Competitive aminal formation during the synthesis of a highly soluble, isopropyl-decorated imine porous organic cage†

**DOI:** 10.1039/d3cc00072a

**Published:** 2023-02-23

**Authors:** Rachel J. Kearsey, Andrew Tarzia, Marc A. Little, Michael C. Brand, Rob Clowes, Kim E. Jelfs, Andrew I. Cooper, Rebecca L. Greenaway

**Affiliations:** a Department of Chemistry and Materials Innovation Factory, University of Liverpool 51 Oxford Street Liverpool L7 3NY UK aicooper@liverpool.ac.uk; b Department of Chemistry, Molecular Sciences Research Hub, Imperial College London 82 Wood Lane London W12 0BZ UK r.greenaway@imperial.ac.uk

## Abstract

The synthesis of a new porous organic cage decorated with isopropyl moieties (CC21) was achieved from the reaction of triformylbenzene and an isopropyl functionalised diamine. Unlike structurally analogous porous organic cages, its synthesis proved challenging due to competitive aminal formation, rationalised using control experiments and computational modelling. The use of an additional amine was found to increase conversion to the desired cage.

## Abstract 2

An imine-derived isopropyl-functionalised POC was synthesised over competitive aminal formation by introducing an additional amine in the assembly process.

## Graphical abstract

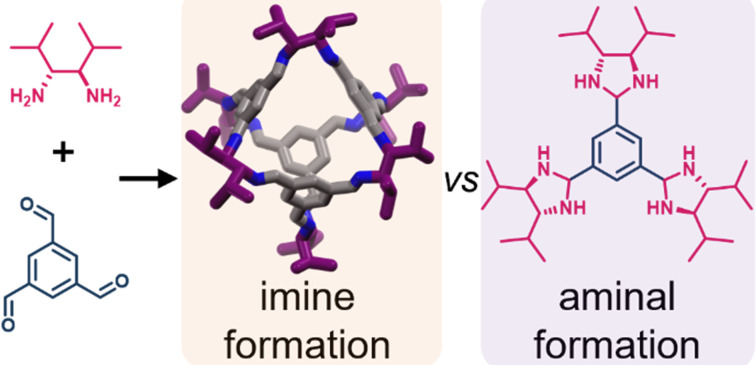

Porous materials, including zeolites, metal–organic frameworks (MOFs), and organic polymers, are emerging in applications such as gas storage, molecular separations, and heterogeneous catalysis.^1,2^ Unlike these framework materials, porous organic cages (POCs) are discrete covalent molecules containing intrinsic pores that can pack together in the solid state to form interconnected porous networks.^3–5^ Due to their molecular and modular nature, the functionality on the outer periphery can be altered, changing both the chemical and physical properties of the resulting cage.^6–8^ Additionally, modification of the external functionality can be used to both direct, or disrupt, the crystal packing, with the latter resulting in amorphous and often more soluble materials.^9,10^

Previously, we have reported the synthesis of POCs formed *via* [4+6] cycloimination reactions – that is, 4 equivalents of a tritopic aldehyde are reacted with 6 equivalents of a ditopic amine, in a 10-component assembly process. Typically this involves the reaction of 1,3,5-triformylbenzene (TFB) with a range of different vicinal diamines.^11–13^ However, some functionality is particularly challenging to incorporate. For example, ditopic vicinal diamines decorated with aromatic groups,^14^ tethered macrocycles,^15^ and long alkyl functionality,^16^ tend to require harsher reaction conditions and often lead to lower yields of the desired cage, which can also be unstable.

The dynamic covalent reversibility of imine condensations, and the modular nature of cage synthesis, can also be utilised to improve certain properties. For example, many POCs tend to have relatively poor solubility in organic solvents, despite their molecular nature making them solution processable. Dynamic covalent scrambling has been shown to dramatically increase POC solubility.^10^ This involves reacting more than one vicinal diamine with TFB, to form a statistical distribution of different periphery functionalised cages, without affecting the tetrahedral cage structure. Usually, scrambling is favoured over both self-sorting into the two parent cages or social self-sorting into a new three-component species; in the majority of cases where a mixture of vicinal diamines is used, the outcome is a statistical distribution of POCs, no matter what the diamine feed ratio is.^10,17^ In more recent studies using high-throughput robotics to automate the synthesis of scrambled POC mixtures,^18^ it was found that mixtures of vicinal diamines did not always lead to the formation of a statistical distribution of cage species. In particular, one combination involving 2-methylpropane-1,2-diamine (MPDA, used in the synthesis of CC13,^19^ 1 equiv.) and (3*R*,4*R*)-2,5-dimethylhexane-3,4-diamine (DMHDA, 5 equiv.) led to the isolation of a novel isopropyl decorated parent cage, CC21, albeit in a low 5% yield (Fig. 1a).^18^ When the direct synthesis of CC21 was then attempted using 6 equivalents of DMHDA in CHCl_3_, and in the absence of MPDA, the parent cage was not isolated. Instead, an alternative tri-aminal side-product (1,3,5-tris(4,5-diisopropyl-imidazolidin-2-yl)benzene, Fig. 1b) was formed and isolated in 33% yield. These preliminary results prompted us to carry out further investigations into the formation of CC21. Here, we report the synthesis, characterisation, and investigation into the formation and properties of this new POC, which proved to be both highly soluble and have unusual crystal packing, likely due to the presence of the sterically bulky isopropyl functionalities on the cage vertices.

**Fig. 1:**
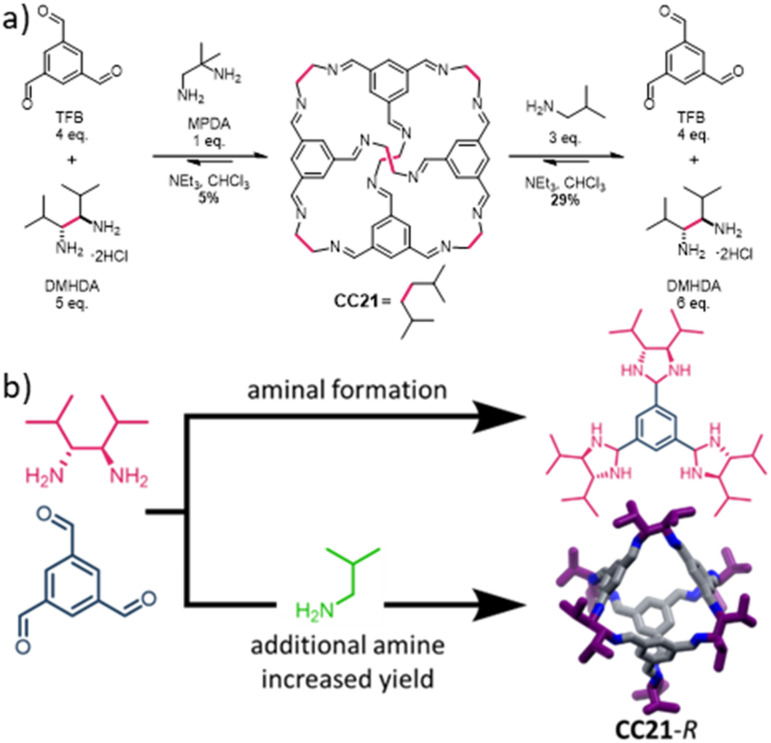
(a) Synthesis of CC21 – a Tri^4^Di^6^ porous organic cage formed through the dynamic imine condensation of 1,3,5-triformylbenzene (TFB) with (3*R*,4*R*)-2,5-dimethylhexane-3,4-diamine or (3*S*,4*S*)-2,5-dimethylhexane-3,4-diamine (*R*,*R*- or *S*,*S*-DMHDA) in the presence of an additional amine (either 2-methylpropane-1,2-diamine (MPDA) or isobutylamine); (b) schematic of the effect of reaction conditions on the formation of CC21 or aminal intermediates.

First, a series of ^1^H NMR experiments were carried out probing the neat reaction between TFB and DMHDA, and the reaction in the presence of the ditopic amine MPDA or a mono-amine, namely isobutylamine, which would avoid the formation of a competing cage and avoid the risk of scrambling occurring (ESI,† Fig. S1, S3 and S5). The reaction mixtures were also analysed by high-resolution mass-spectrometry (HRMS) at different time points to aid in the identification of key reaction intermediates (ESI,† Fig. S2, S4 and S6). In each of the conditions investigated, no insoluble precipitate formed over the course of the reaction, and common intermediates formed from the reaction of TFB with DMHDA could be identified and persisted throughout the reactions (Fig. 2 and ESI,† Tables S1, S5, Fig. S7) – these included [1+2], [1+3], [2+3], [2+4], [3+4], [3+5], and [3+6] intermediates ([TFB + DMHDA]) with varying degrees of condensation and water loss. While a general shift to larger oligomeric species could be observed in the mass spectra over time, complete conversion of the building blocks and oligomeric species to the desired Tri^4^Di^6^ POC, CC21, was not observed even after a prolonged reaction time of 6 weeks, with only small quantities of the [4+6] mass ion apparent in the mass spectra.

**Fig. 2:**
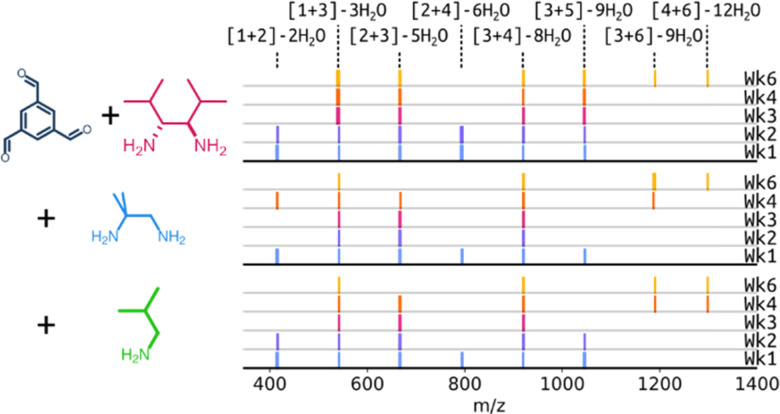
Representation of the mass spectra collected over 6 weeks for the different reaction conditions screened to investigate formation of CC21 in CDCl_3_ with NEt_3_ at room temperature, with the observed identifiable [TFB + DMHDA] intermediates shown. Note that this does not include any information in relation to relative peak intensities in the mass spectra.

To investigate if these identified intermediates were kinetic traps during the reaction and preventing the formation of CC21, we modelled each intermediate and determined their formation energy using GFN2-xTB (see ESI† for details).^20^ This was compared against the same intermediates for other structurally analogous and previously synthesised cages, in particular CC1 (formed using ethylenediamine, EDA), CC3 (formed using cyclohexyldiamine, CHDA) and CC13 (formed using MPDA), to evaluate if there were significant differences that might explain the observed behaviour in CC21 formation (Fig. 3). We do not model the barriers between intermediates, which are a key factor in determining the influence of kinetic trapping. To extract the numerous possible transition states for these systems (the formation of the cage requires 12 imine bonds to be formed) would be too computationally expensive and is therefore beyond our study here. Knowledge of the reaction barriers is not necessary for the analysis proposed here, which is similar to that reported by Zhu *et al.*^21^ Overall, the general trends across the reaction pathways were comparable for all four cages (Fig. 3a), suggesting that the formation of CC21 should be theoretically viable based purely on imine-derived intermediates and the successful formation of the other three cages. Even though we used a semiempirical method, the trend we obtain in formation energies agree with those of Sholl and co-workers.^21^ However, given the isolation and identification of the tri-aminal species in the preliminary studies (Fig. 1b), and the fact that the identified intermediates from the HRMS could also correspond to intramolecularly cyclised aminals (ESI,† Fig. S7), it was theorised that perhaps the formation of competing aminal intermediates were acting as traps along the reaction pathway to CC21. Therefore, a series of control reactions were carried out between the different di-topic amines (DMHDA, EDA, CHDA, & MPDA) and benzaldehyde, alongside further investigation of the formation energies for the aminal derivatives of the intermediates for CC21.

**Fig. 3:**
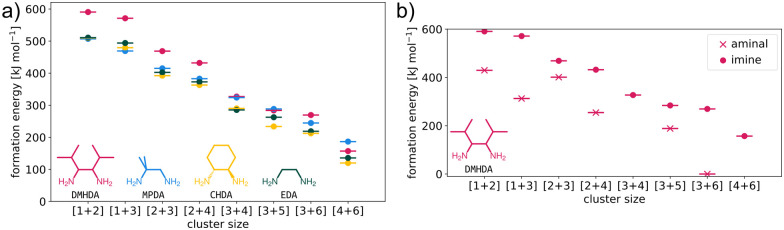
(a) Comparison of the formation energies for a range of imine intermediates on the pathway to the formation of CC1 (EDA), CC3 (CHDA), CC13 (MPDA), and CC21 (DMHDA). Data is coloured based on the inset amines. (b) Comparison of the formation energies for the aminal (pink crosses) and imine (pink circles) intermediates identified during the formation of CC21.

In the series of control reactions (Fig. 4a), when either 1 or 2 equivalents of benzaldehyde were reacted with EDA, CHDA or MPDA, the mono- or bis-imines were formed, respectively (ESI,† Fig. S8–S10). However, when DMHDA was used, clean conversion to the aminal was observed in the presence of 1 equivalent of benzaldehyde, and a mixture of aminal, residual aldehyde, and partial imine formation was observed in the presence of 2 equivalents (ESI,† Fig. S11). The preference for this vicinal diamine to form aminals over di-imines can be further rationalised by looking at the preferred configurations of the different diamines. Therefore, we studied the distribution of structural parameters of the four diamines (EDA, CHDA, MPDA and DMHDA) using the conformer generation algorithm ETKDG (in the *RDKit* software)^22,23^ to generate 500 conformers, and the semiempirical density functional tight-binding method, GFN2-xTB,^20^ to geometry optimise each conformer. We suggest that the distribution of the N–C–C–N dihedral angles and N–N distances for the four amines will infer their preference for aminal reactivity, where dihedrals nearer 0° and shorter N–N distances imply better kinetics for aminal formation due to the closer proximity of the N atoms. Fig. 4c shows the distribution of the N–C–C–N dihedral angles over the conformers are within 10 kJ mol^−1^ of the lowest energy conformer, where the preference for close N–N positioning in DMHDA is clearly shown. Fig. S26 (ESI†) shows the complete set of N–N distance and N–C–C–N dihedral data. This can also be visualised with Newman projections (Fig. 4b) – the steric bulk of the isopropyl functionality likely forces the amines closer together compared to MPDA and EDA and the highly pre-configured CHDA with its chair conformation. This closer proximity of the amines is expected to favour aminal formation kinetics. In addition, a comparison of the formation energies for the aminal intermediates *versus* the imine analogues (Fig. 3b) clearly shows that the aminal intermediates would be thermodynamically preferred over the imine intermediates, subsequently preventing conversion to CC21.

**Fig. 4:**
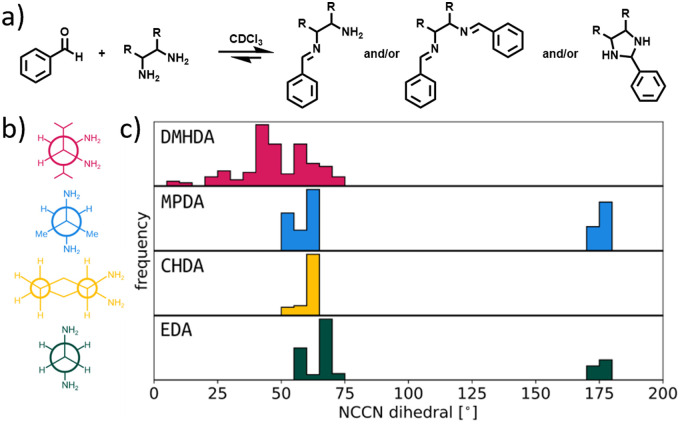
(a) Series of control reactions to probe the preference of different diamines to form either imines or aminals. (b) Newman projections of the four diamines used in this study, shown as the least sterically hindered conformers. (c) N–C–C–N dihedral distributions for conformers within 10 kJ mol^−1^ of the lowest energy conformer for the four diamines. Colours match (b).

Based on this knowledge, we hypothesised that the presence of an additional diamine in the initial studies^18^ promoted the formation of CC21 by acting as an intermediary species to disrupt the aminal reaction pathway. Therefore, a series of reactions was carried out to determine if formal transimination was occurring, a strategy previously applied in the synthesis of imine-linked covalent organic frameworks (COFs),^24^ and to investigate if an increased quantity of an additional mono-amine would improve conversion to the desired POC (see ESI† for further details). Interestingly, when a large excess of isobutylamine (12.0 equiv.), or a pre-formed tri-imine intermediate from the reaction of TFB with isobutylamine was used as a formal transimination precursor, a ‘scrambled’ statistical distribution of tritopic species containing both isobutylamine and DMHDA was formed (ESI,† Fig. S15 and S16), analogous to the side-product formed on direct reaction of TFB with DMHDA (Fig. 1b), rather than the desired CC21. As might be expected, the use of 1 equivalent of isobutylamine in the presence of either 5 or 6 equivalents of DMHDA mainly led to the same oligomer distribution observed in the initial screen (Fig. 2). In contrast, the use of 3 equivalents of isobutylamine with 6 equivalents of DMHDA and 4 equivalents of TFB led to CC21 being isolated in a much more reasonable 29% yield after 14 days (ESI,† Fig. S17–S20). This suggests that a balance is required in relation to the quantity of additional mono-amine used, with enough amine present to drive formation towards the desired cage species, but not such a large excess that would result in the formation of a thermodynamic statistical distribution of products.

Our attention then turned to investigating the properties of the new cage. First, the solubility of CC21 was investigated since the flexibility and size of the isopropyl groups on the periphery would potentially increase the disorder in the bulk packing and improve the solubility compared to other previously studied structurally analogous POCs. Although imine cages are inherently solution processable due to their discrete molecular nature, they generally have quite low solubilities. The solubility of CC21 in CHCl_3_ was found to be slightly lower than CC13, the most soluble derivative of these cage analogues found to date (165 *vs.* 200 mg mL^−1^, respectively), but this is still a drastic improvement over most of the CC-family (for example, CC3 has a solubility of 9 mg mL^−1^ in CHCl_3_), suggesting it may be suitable for applications requiring high solubility such as porous liquids.^18,25^

Solvated crystals of chirally pure CC21-*R* that were suitable for single crystal X-ray diffraction (SCXRD) were obtained by slowly diffusing MeOH into a solution of CC21 dissolved in CHCl_3_. In the solvated crystal structure, CC21·CHCl_3_·MeOH, the packing of the CC21 cages is frustrated by the isopropyl groups on the cage vertices, which creates extrinsic voids that are full of disordered solvent molecules. In this study, we also re-crystallised CC21-S from CH_2_Cl_2_ by slowly evaporating a saturated solution at room temperature. By comparison, the crystalline material isolated from CH_2_Cl_2_ was activated at 363 K under a dynamic vacuum before being analysed by single crystal and powder X-ray diffraction (PXRD). After activation under these conditions, we determined the structure of a new phase, CC21α, which is structurally related to CC21·CHCl_3_·MeOH but has a unit cell volume that is 15% smaller (ESI,† Table S2) due to a denser packing of CC21 molecules. Notably, the PXRD pattern for the activated bulk sample crystallised from CH_2_Cl_2_ matches the simulated PXRD of CC21α (ESI,† Fig. S23), and we used this sample for N_2_ gas sorption analysis at 77.3 K (ESI,† Fig. S24). CC21α was found to adsorb 11.1 mmol g^−1^ of N_2_ at 1 bar and 77.3 K, and has a Brunauer–Emmett–Teller (BET) surface area of 699 m^2^ g^−1^. PXRD data recorded post gas sorption analysis revealed that CC21α did not change structure during this measurement (ESI,† Fig. S23), indicating that the bulky isopropyl groups do not affect the stability of the activated material. This data also proves that CC21 is shape persistent in the solid state and retains its cavity in the absence of solvent. Computational assessment of the porosity of the obtained crystal structure suggests that channels through the structure are approximately the size of an N_2_ molecule, which allows for nitrogen adsorption (ESI,† Fig. S29).

In conclusion, the synthesis of a highly soluble porous organic cage, CC21, has been achieved through the introduction of additional amines during the cage self-assembly. Investigation of the prominent reaction intermediates and of control reactions found that cage formation was inhibited by the preferred formation of aminal-based species. Computational analysis of these intermediates for four diamines (DMHDA and three cage-forming diamines) found similar qualitative trends in formation energies toward cage formation. However, where possible, the aminal species was found to be more stable than the imine species. We show that DMHDA favours conformations more amenable to aminal formation than the three other diamines. Therefore, we suggest a stronger preference for forming the aminal species for DMHDA compared to the other diamines, which hinders cage formation. From this, we explored reaction conditions and the use of an additional mono-amine to optimise the cage-formation reaction, which demonstrated the importance of balancing transimination towards the desired cage species with the formation of distinct self-assembled species containing both DMHDA and the additional amine.

The authors thank the Engineering and Physical Sciences Research Council (EPSRC) under the Grants EP/R005710/1 and EP/W01601X/1 for financial support. R. L. G. and K. E. J. thank the Royal Society for University Research Fellowships. We acknowledge funding from the European Research Council under FP7 (CoMMaD, ERC Grant No. 758370) and the Royal Society for an Enhancement Award 2018. The authors acknowledge the MicroBioRefinery and the Agilent Measurement Suite for assistance with QTOF-MS measurements. This work used the ARCHER2 UK National Supercomputing Service (https://www.archer2.ac.uk) *via* the UK's HEC Materials Chemistry Consortium, which is funded by the EPSRC (EP/L000202, EP/R029431, EP/T022213). For the purpose of open access, the author has applied a Creative Commons Attribution (CC BY) licence to any Author Accepted Manuscript version arising.

## Conflicts of interest

There are no conflicts to declare.

## Supplementary Material

CC-059-D3CC00072A-s001

CC-059-D3CC00072A-s002
