# Supplementary material for: Competitive aminal formation during the synthesis of a highly soluble, isopropyl-decorated imine porous organic cage
Source: Chem Commun (Camb). 2023 Feb 23;59(25):3731–4. doi: 10.1039/d3cc00072a (PMC10035065; doi:10.1039/d3cc00072a)
Supplement: CC-059-D3CC00072A-s001 [file CC-059-D3CC00072A-s001.pdf]

Electronic Supplementary Material (ESI) for ChemComm.
This journal is © The Royal Society of Chemistry 2023

# Supporting Information

## Competitive Aminal Formation during the Synthesis of a Highly Soluble, Isopropyl-Decorated Imine Porous Organic Cage

Rachel J. Kearsey,[a] Andrew Tarzia,[b] Marc A. Little,[a] Michael C. Brand,[a] Rob Clowes,[a] Kim E. Jelfs,[b] Andrew I. Cooper*[a] and Rebecca L. Greenaway*[b]

[a]Department of Chemistry and Materials Innovation Factory, University of Liverpool, 51 Oxford Street, Liverpool, L7 3NY, UK.

[b]Department of Chemistry, Molecular Sciences Research Hub, Imperial College London, 82 Wood Lane, London, W12 0BZ, UK.

Emails: r.greenaway@imperial.ac.uk; aicooper@liverpool.ac.uk

# 1. General synthetic and analytical methods

**Materials:** 1,3,5-Triformylbenzene (TFB) was purchased from Manchester Organics (UK). Other chemicals were purchased from Fluorochem UK, TCI UK or Sigma-Aldrich. Solvents were reagent or HPLC grade purchased from Fischer Scientific. All materials were used as received unless stated otherwise.

**Synthesis:** All reactions were stirred magnetically using Teflon-coated stirrer bars. Where heating was required, the reactions were warmed using a stirrer hotplate with heating blocks, with the stated temperature being measured externally to the reaction flask with an attached probe. Removal of solvents was done using a rotary evaporator.

**IR:** Infra-red (IR) spectra were recorded on a Bruker Tensor 27 FT-IR using ATR measurements for oils and solids as neat samples or using transmission mode on a 96-well silica wafer deposited as a thin film as part of the high-throughput analysis.

**NMR:** $^1$H Nuclear magnetic resonance (NMR) spectra were recorded using an internal deuterium lock for the residual protons in $CDCl_3$ (δ = 7.26 ppm), $D_2O$ (δ = 4.79 ppm), or $CD_2Cl_2$ (δ = 5.32 ppm) at ambient probe temperature on either a Bruker Avance 400 (400 MHz) or Bruker DRX500 (500 MHz) spectrometer. Data presented as follows: chemical shift, integration, peak multiplicity (s = singlet, d = doublet, t = triplet, q = quartet, m = multiplet, br = broad) and coupling constants (J / Hz). Chemical shifts are expressed in ppm on a δ scale relative to $\delta_{TMS}$ (0 ppm), $\delta_{D2O}$ (4.79 ppm), $\delta_{CD2Cl2}$ (5.32 ppm), or $\delta_{CDCl3}$ (7.26 ppm). $^{13}$C NMR Spectra were recorded using an internal deuterium lock using $CDCl_3$ (δ = 77.16 ppm) at ambient probe temperatures on the following instruments: Bruker Avance 400 (101 MHz) or Bruker DRX500 (126 MHz).

**HPLC:** HPLC analysis was carried out using a Dionex UltiMate 3000 with a diode array UV detector using a Thermo-Scientific Syncronis C8 column, 150 x 4.6 mm, 3 µm (SN 10136940, Lot 12459). The mobile phase was isocratic MeOH at a flow rate of 1 mL/min for a 10-30 min run time, and the column temperature was set to 30 °C. The injection volume was 10 µL and the sample concentration was approximately 1 mg/mL. Detection for UV analysis was conducted at 254 nm.

**HRMS:** High resolution mass spectrometry (HRMS) was carried out using an Agilent Technologies 6530B accurate-mass QTOF Dual ESI mass spectrometer (capillary voltage 4000 V, fragmentor 225 V) in positive-ion detection mode. The mobile phase was MeOH + 0.1% formic acid at a flow rate of 0.25 mL/min.

**PXRD:** PXRD patterns were collected in transmission mode on samples held on thin Mylar film in aluminium well plates on a Panalytical Empyrean diffractometer, equipped with a high throughput screening XYZ stage, X-ray focusing mirror, and PIXcel detector, using Cu-Kα (λ = 1.541 Å) radiation. PXRD patterns were recorded at room temperature over the $2\theta$ range 1–56°, in 0.013° steps, for 30 minutes.

**Single crystal X-ray Crystallography:** SC-XRD data sets were measured on a Rigaku MicroMax-007 HF rotating anode diffractometer (Mo-Kα radiation, λ = 0.71073 Å, Kappa 4-circle goniometer, Rigaku Saturn724+ detector). Structures were solved with SHELXT[1] and refined by full-matrix least squares on $|F|^2$ by SHELXL,[2] interfaced through the programme OLEX2.[3] Absolute configuration was based on experimental synthetic procedures. Due to solvent disorder in the crystal structure, 2(**CC21**)·9($CHCl_3$)·10.5($CH_4O$)·(H2O), the $CHCl_3$ and MeOH solvent molecules were refined with bond distance restraints (DFIX and DANG in SHELX) and rigid bond restraints (RIGU in SHELX).

**Gas sorption analysis:** Surface areas were measured by nitrogen sorption at 77.3 K. Powder samples were degassed on the analysis port under vacuum. Isotherm measurements were performed using a Micromeritics 3flex surface characterization analyzer, equipped with a Cold-Edge technologies liquid helium cryostat chiller unit for temperature control.

## 2. Identification of Reaction Intermediates – $^1$H NMR & HRMS Studies

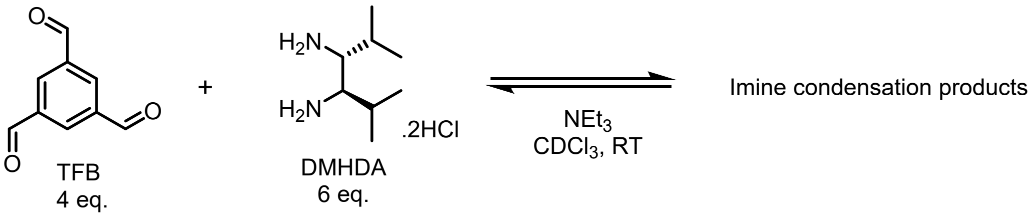


1,3,5-Triformylbenzene (0.033 g, 0.206 mmol, 4 eq.) was dissolved in $CDCl_3$ (3 mL), and (3*R*,4*R*)-2,5-dimethylhexane-3,4-diamine dihydrochloride (0.067 g, 0.308 mmol, 6 eq.) and triethylamine (0.14 mL, 1.02 mmol, 3.3 eq.) in $CDCl_3$ (3 mL), was added. The reaction was set to stir at room temperature and monitored using $^1$H NMR spectroscopy and high-resolution mass spectroscopy (HRMS).

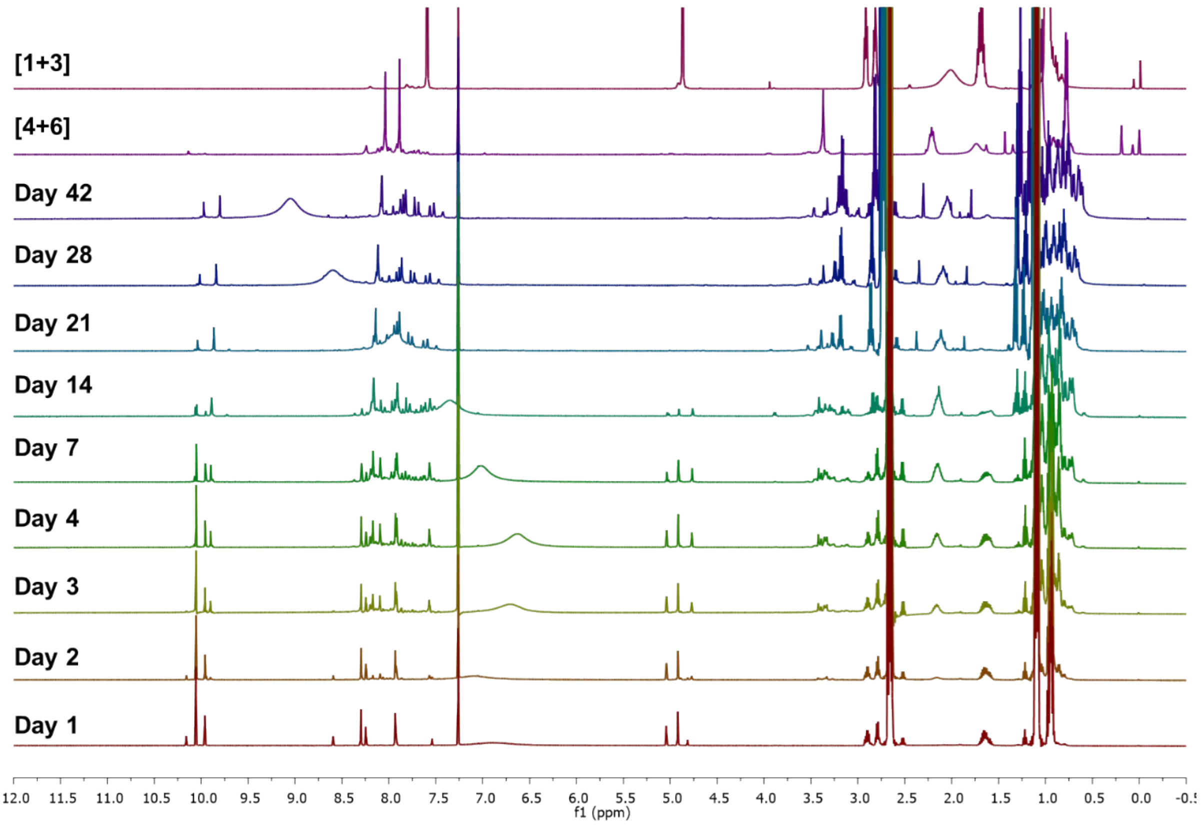


**Figure S1:** Stacked $^1$H NMR spectra ($CDCl_3$) for the tracked reaction between 1,3,5-triformylbenzene and (3*R*,4*R*)-2,5-dimethylhexane-3,4-diamine over time compared to the isolatable [1+3] intermediate 1,3,5-tris((4*R*,5*R*)-4,5-diisopropylimidazolidin-2-yl)benzene and the [4+6] porous organic cage **CC21**.

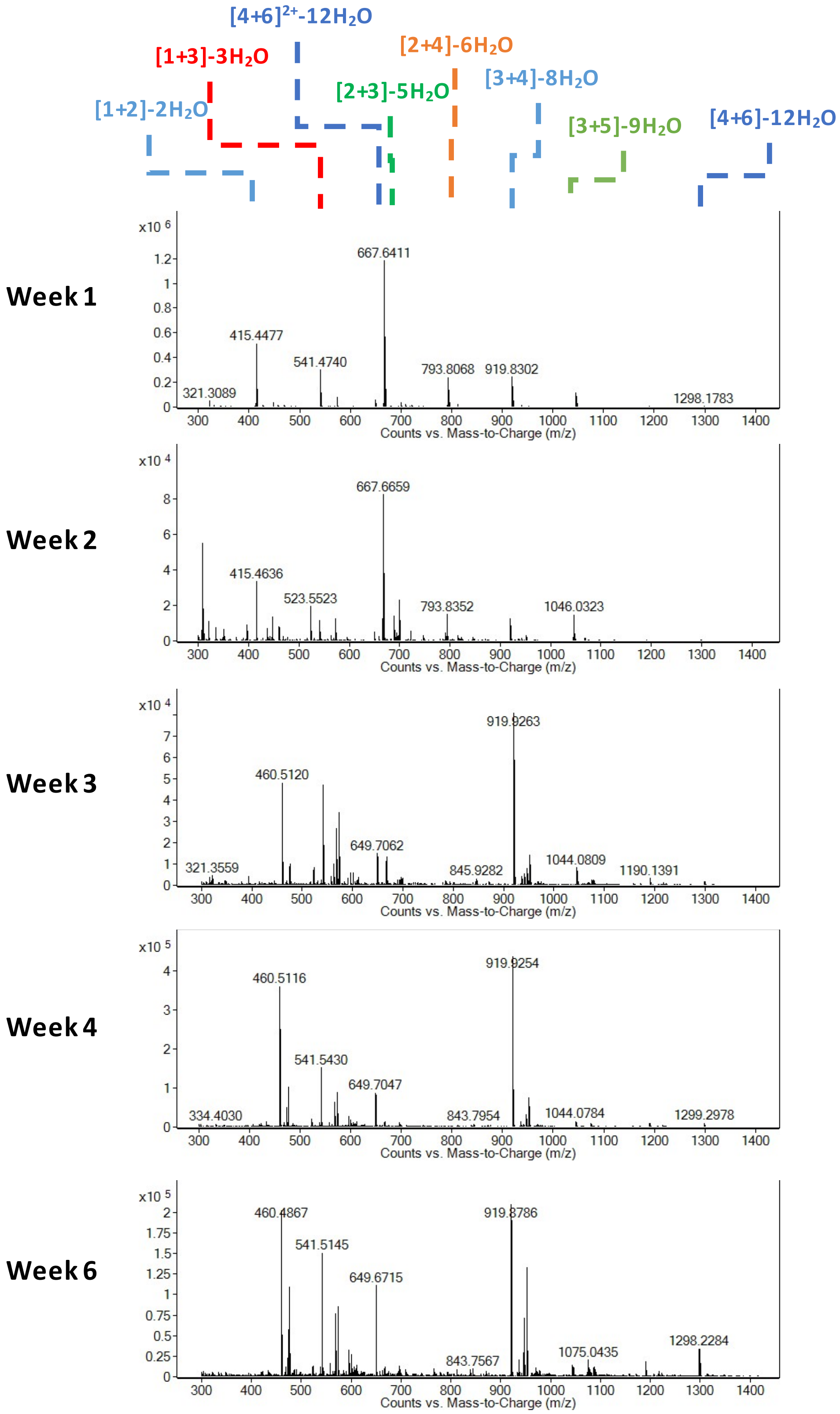


**Figure S2:** Stacked HRMS spectra for the tracked reaction between 1,3,5-triformylbenzene and (3*R*,4*R*)-2,5-dimethylhexane-3,4-diamine over time, monitored periodically to identify the intermediates present.

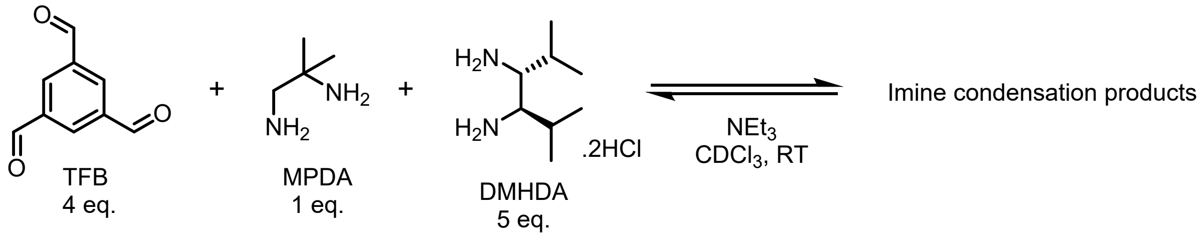


1,3,5-Triformylbenzene (33 mg, 0.2055 mmol, 4 eq.) was dissolved in $CDCl_3$ (3 mL), and 2-methylpropane-1,2-diamine (4.5 mg, 0.0514 mmol, 1 eq.) in $CDCl_3$ (1.5 mL), and (3*R*,4*R*)-2,5-dimethylhexane-3,4-diamine dihydrochloride (5.9 mg, 0.257 mmol, 5 eq.) in $CDCl_3$ (1.5 mL) with triethylamine (0.12 mL, 0.848 mmol, 3.3 eq.), were added. The reaction was set to stir at room temperature and monitored using $^1H$ NMR spectroscopy and high-resolution mass spectroscopy (HRMS).

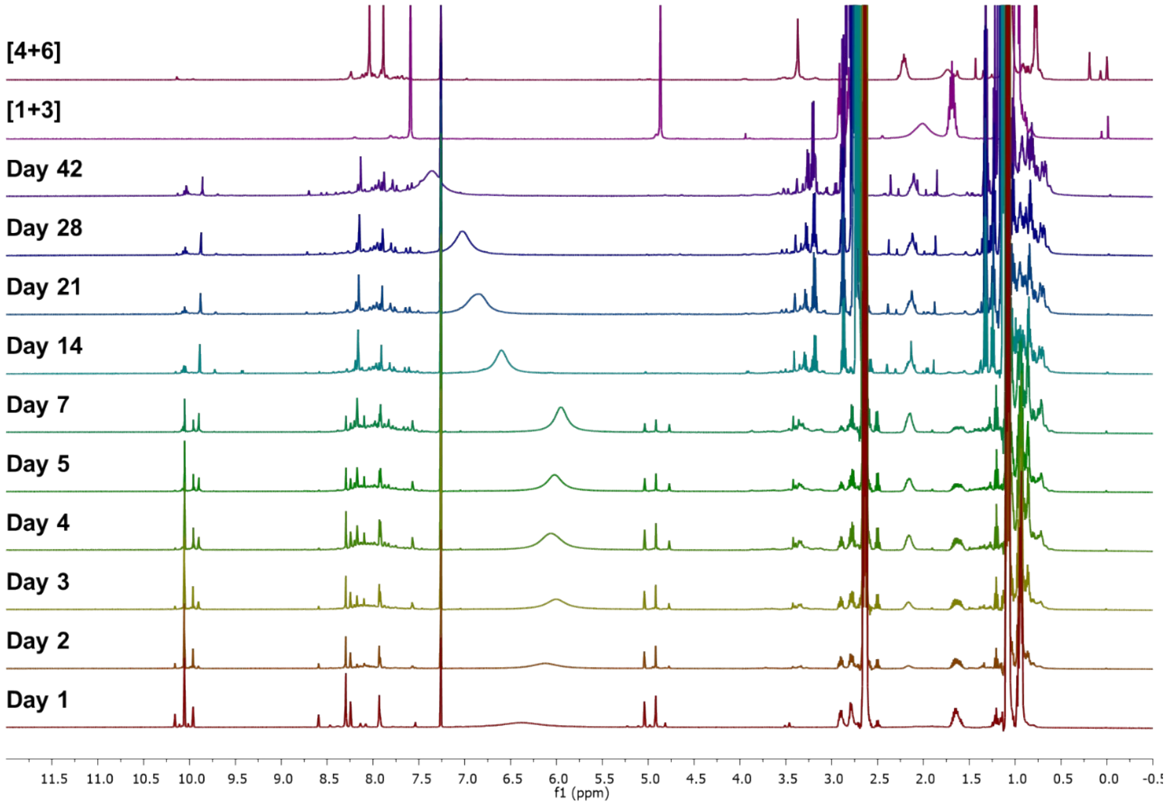


**Figure S3:** Stacked $^1H$ NMR spectra ($CDCl_3$) tracking the reaction between 1,3,5-triformylbenzene, (*3R,4R)*-2,5-dimethylhexane-3,4-diamine, and 1,2-diamino-2-methylpropane over time compared to the isolatable [1+3] intermediate 1,3,5-tris((4R,5R)-4,5-diisopropylimidazolidin-2-yl)benzene and the [4+6] porous organic cage **CC21**.

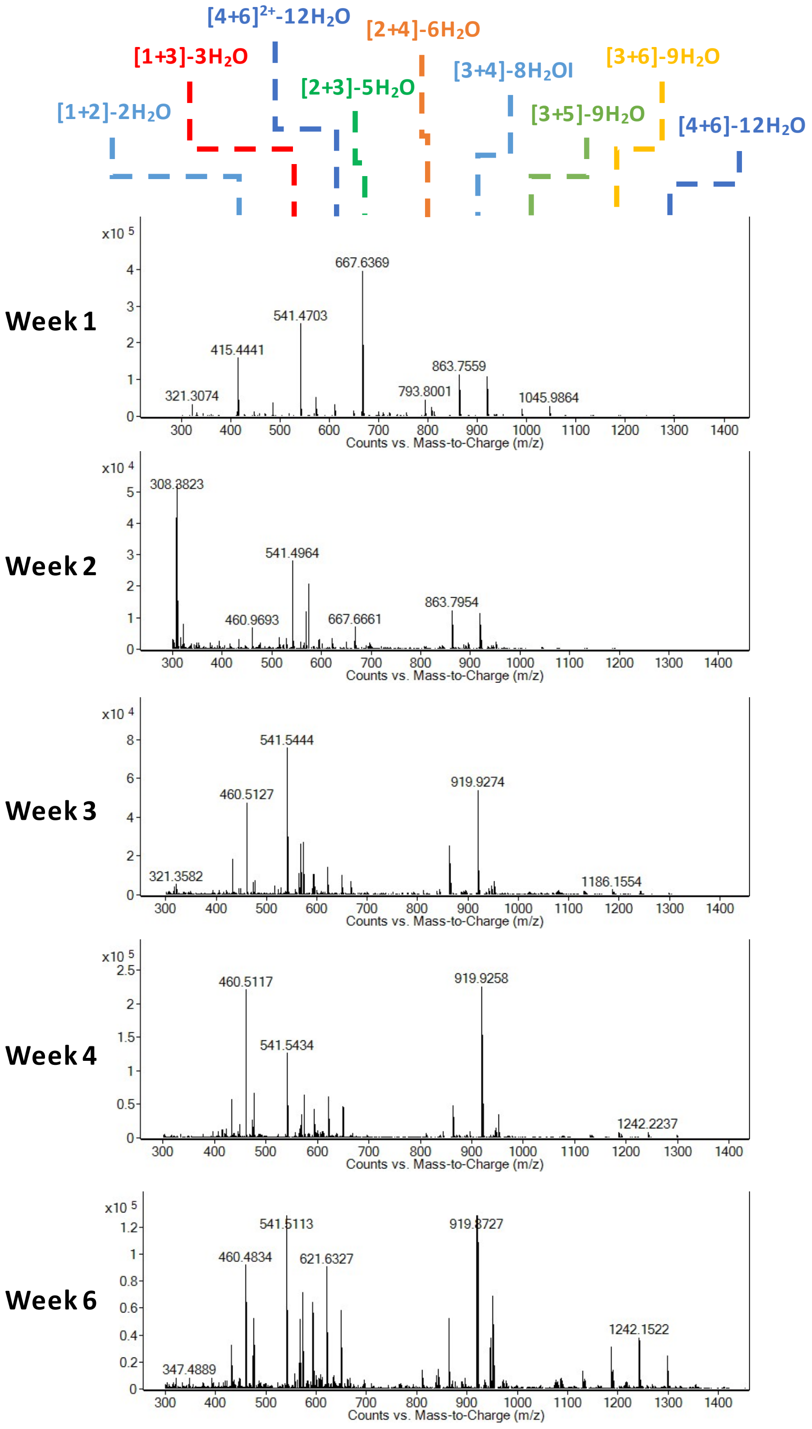


**Figure S4:** Stacked HRMS spectra for the tracked reaction between 1,3,5-triformylbenzene, (3*R*,4*R*)-2,5-dimethylhexane-3,4-diamine, and 1,2-diamino-2-methylpropane over time, monitored periodically to identify the intermediates present.

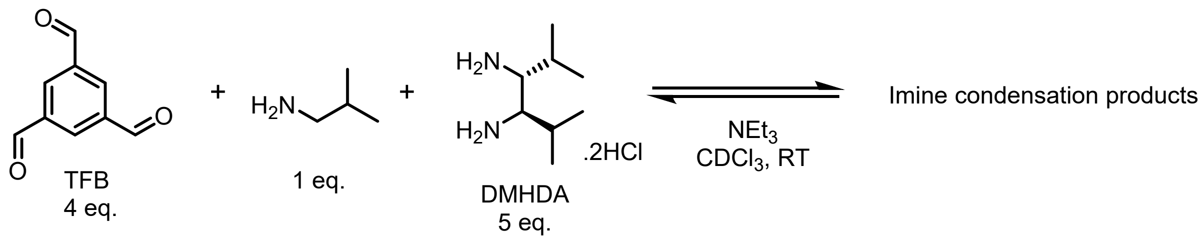


1,3,5-Triformylbenzene (37 mg, 0.226, 4 eq.) was dissolved in $CDCl_3$ (3 mL), and isobutylamine (4.1 mg, 0.0566 mmol, 1 eq.) in $CDCl_3$ (1.5 mL), and (3*R*,4*R*)-2,5-dimethylhexane-3,4-diamine dihydrochloride (61.5 mg, 0.283 mmol, 5 eq.) in $CDCl_3$ (0.13 mL, 0.934 mmol, 3.3 eq.) with triethylamine (0.02 mL, 0.17 mmol, 3.3 eq.), were added. The reaction was set to stir at room temperature and monitored using $^1$H NMR spectroscopy and high-resolution mass spectroscopy (HRMS).

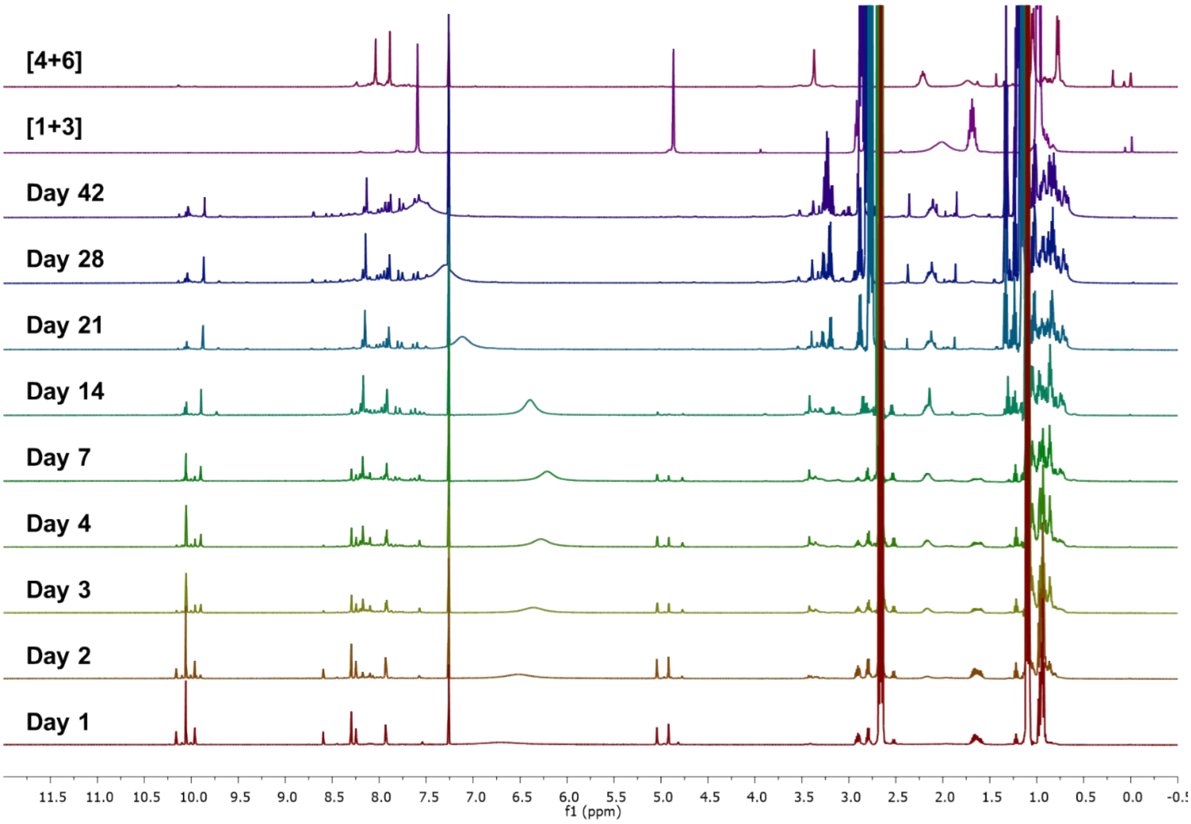


**Figure S5:** Stacked $^1$H NMR spectra ($CDCl_3$) tracking the reaction between 1,3,5-triformylbenzene and (*3R*,4*R)*-2,5-dimethylhexane-3,4-diamine with isobutylamine over time compared to the isolatable [1+3] intermediate 1,3,5-tris((4R,5R)-4,5-diisopropylimidazolidin-2-yl)benzene and the [4+6] porous organic cage **CC21**.

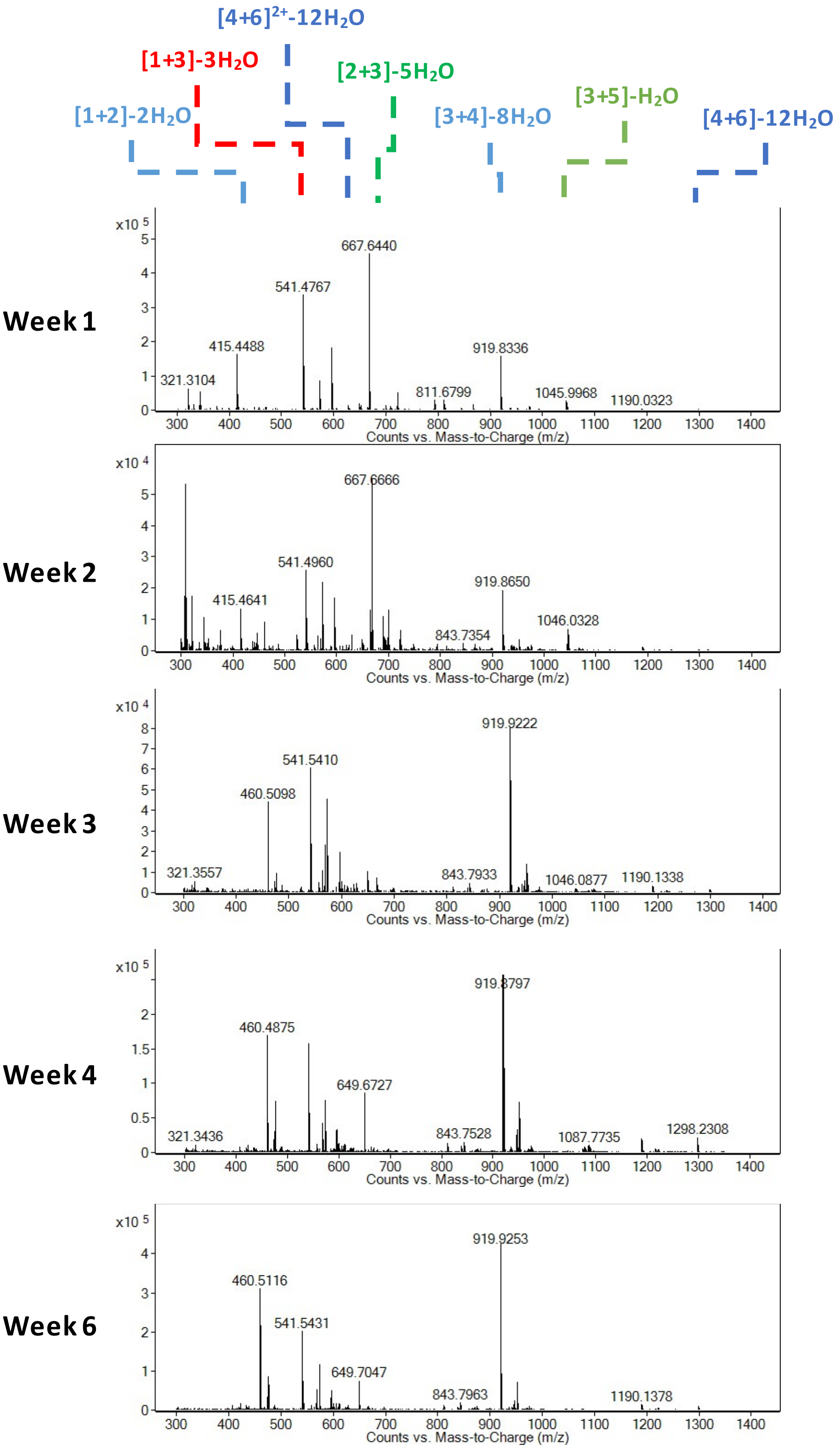


**Figure S6:** Stacked HRMS spectra for the tracked reaction between 1,3,5-triformylbenzene and (3*R*,4*R*)-2,5-dimethylhexane-3,4-diamine with isopropylamine over time, monitored periodically to identify the intermediates present.

**Table S1:** Possible intermediates during the formation of **CC21**

| Entry | No. of TFB | No. of DMHDA | No. of Imine Bonds | MW (calc.) | m/z (calc.) | calc. $[M+H]^{+}$ | calc. $[M+2H]^{2+}$ | calc. $[M+Na]^{+}$ | calc. $[M+2Na]^{2+}$ | calc. $[M+H+Na]^{2+}$ |
|---|---|---|---|---|---|---|---|---|---|---|
| **1** | 1 | 1 | 1 | 288.3910 | 288.1837 | 289.1915 | 145.09965 | 311.1735 | 167.08165 | 156.0907 |
| **2** | 1 | 2 | 2 | 414.6380 | 414.3357 | 415.3435 | 208.17565 | 437.3255 | 207.16785 | 219.1667 |
| **3** | 1 | 3 | 3 | 540.8850 | 540.4877 | 541.4955 | 271.25165 | 563.4775 | 270.24385 | 282.2427 |
| **4** | 2 | 1 | 2 | 432.5200 | 432.2048 | 433.2126 | 217.1102 | 455.1946 | 216.1024 | 228.1012 |
| **5** | 2 | 2 | 3 | 558.7670 | 558.3568 | 559.3646 | 280.1862 | 581.3466 | 279.1784 | 291.1772 |
| **6** | 2 | 2 | 4 | 540.7520 | 540.3462 | 541.3540 | 271.1809 | 563.3360 | 270.1731 | 282.1719 |
| **7** | 2 | 3 | 4 | 685.0140 | 684.5088 | 685.5166 | 343.2622 | 707.4986 | 342.2544 | 354.2532 |
| **8** | 2 | 3 | 5 | 666.9990 | 666.4982 | 667.5060 | 334.2569 | 689.4880 | 333.2491 | 345.2479 |
| **9** | 2 | 3 | 6 | 648.9840 | 648.4876 | 649.4954 | 325.2516 | 671.4774 | 324.2438 | 336.2426 |
| **10** | 2 | 4 | 5 | 811.2610 | 810.6608 | 811.6686 | 406.3382 | 833.6506 | 405.3304 | 417.3292 |
| **11** | 2 | 4 | 6 | 793.2460 | 792.6502 | 793.6580 | 397.3329 | 815.6400 | 396.3251 | 408.3239 |
| **12** | 2 | 5 | 6 | 937.5080 | 936.8128 | 937.8206 | 469.4142 | 959.8026 | 468.4064 | 480.4052 |
| **13** | 3 | 2 | 4 | 702.8960 | 702.3779 | 703.3857 | 352.19675 | 725.3677 | 351.18895 | 363.1878 |
| **14** | 3 | 3 | 5 | 829.1430 | 828.5299 | 829.5377 | 415.27275 | 851.5197 | 414.26495 | 426.2638 |
| **15** | 3 | 3 | 6 | 811.1280 | 810.5193 | 811.5271 | 406.26745 | 833.5091 | 405.25965 | 417.2585 |
| **16** | 3 | 4 | 6 | 955.3900 | 954.6819 | 955.6897 | 478.34875 | 977.6717 | 477.34095 | 489.3398 |
| **17** | 3 | 4 | 7 | 937.3750 | 936.6713 | 937.6791 | 469.34345 | 959.6611 | 468.33565 | 480.3345 |
| **18** | 3 | 4 | 8 | 919.3600 | 918.6607 | 919.6685 | 460.33815 | 941.6505 | 459.33035 | 471.3292 |
| **19** | 3 | 5 | 7 | 1081.6370 | 1080.8339 | 1081.8417 | 541.42475 | 1103.8237 | 540.41695 | 552.4158 |
| **20** | 3 | 5 | 8 | 1063.6220 | 1062.8233 | 1063.8311 | 532.41945 | 1085.8131 | 531.41165 | 543.4105 |
| **21** | 3 | 5 | 9 | 1045.6070 | 1044.8127 | 1045.8205 | 523.41415 | 1067.8025 | 522.40635 | 534.4052 |
| **22** | 3 | 6 | 8 | 1207.8840 | 1206.9859 | 1207.9937 | 604.50075 | 1229.9757 | 603.49295 | 615.4918 |
| **23** | 3 | 6 | 9 | 1189.8690 | 1188.9753 | 1189.9831 | 595.49545 | 1211.9651 | 594.48765 | 606.4865 |
| **24** | 3 | 7 | 9 | 1334.1310 | 1333.1379 | 1334.1457 | 667.57675 | 1356.1277 | 666.56895 | 678.5678 |
| **25** | 4 | 3 | 6 | 973.2720 | 972.5510 | 973.5588 | 487.2833 | 995.5408 | 486.2755 | 498.2743 |
| **26** | 4 | 4 | 7 | 1099.5190 | 1098.7030 | 1099.7108 | 550.3593 | 1121.6928 | 549.3515 | 561.3503 |
| **27** | 4 | 4 | 8 | 1081.5040 | 1080.6924 | 1081.7002 | 541.354 | 1103.6822 | 540.3462 | 552.345 |
| **28** | 4 | 5 | 8 | 1225.7660 | 1224.8550 | 1225.8628 | 613.4353 | 1247.8448 | 612.4275 | 624.4263 |
| **29** | 4 | 5 | 9 | 1207.7510 | 1206.8444 | 1207.8522 | 604.43 | 1229.8342 | 603.4222 | 615.421 |
| **30** | 4 | 5 | 10 | 1189.7360 | 1188.8338 | 1189.8416 | 595.4247 | 1211.8236 | 594.4169 | 606.4157 |
| **31** | 4 | 6 | 9 | 1352.0130 | 1351.0070 | 1352.0148 | 676.5113 | 1373.9968 | 675.5035 | 687.5023 |
| **32** | 4 | 6 | 10 | 1333.9980 | 1332.9964 | 1334.0042 | 667.506 | 1355.9862 | 666.4982 | 678.497 |
| **33** | 4 | 6 | 11 | 1315.9830 | 1314.9858 | 1315.9936 | 658.5007 | 1337.9756 | 657.4929 | 669.4917 |
| **34** | 4 | 6 | 12 | 1297.9680 | 1296.9752 | 1297.9830 | 649.4954 | 1319.9650 | 648.4876 | 660.4864 |
| **35** | 4 | 7 | 10 | 1478.2600 | 1477.1590 | 1478.1668 | 739.5873 | 1500.1488 | 738.5795 | 750.5783 |
| **36** | 4 | 7 | 11 | 1460.2450 | 1459.1484 | 1460.1562 | 730.582 | 1482.1382 | 729.5742 | 741.573 |
| **37** | 4 | 7 | 12 | 1442.2300 | 1441.1378 | 1442.1456 | 721.5767 | 1464.1276 | 720.5689 | 732.5677 |
| **38** | 4 | 8 | 11 | 1604.5070 | 1603.3110 | 1604.3188 | 802.6633 | 1626.3008 | 801.6555 | 813.6543 |
| **39** | 4 | 8 | 12 | 1586.4920 | 1585.3004 | 1586.3082 | 793.658 | 1608.2902 | 792.6502 | 804.649 |
| **40** | 4 | 9 | 12 | 1730.7540 | 1729.4630 | 1730.4708 | 865.7393 | 1752.4528 | 864.7315 | 876.7303 |

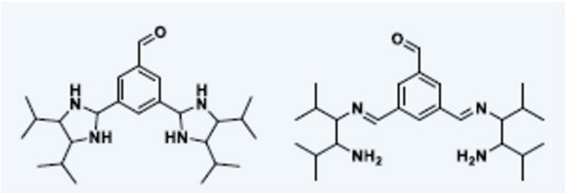

[1+2]

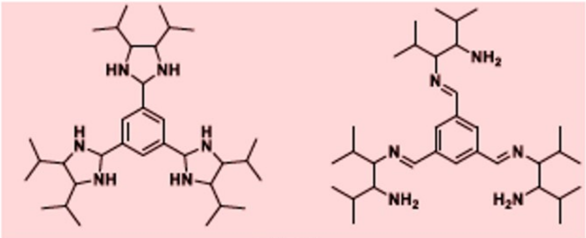

[1+3]

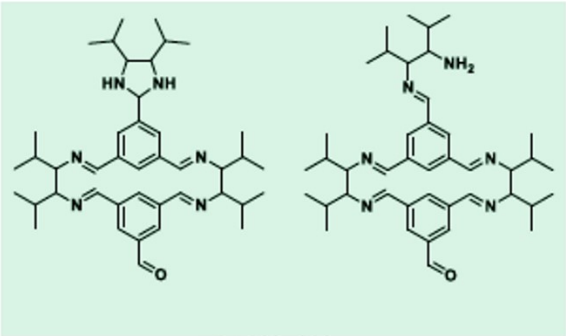

[2+3]

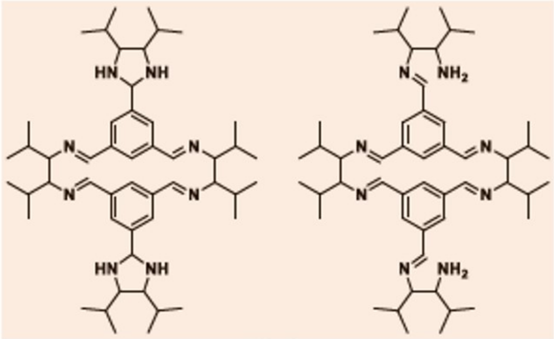

[2+4]

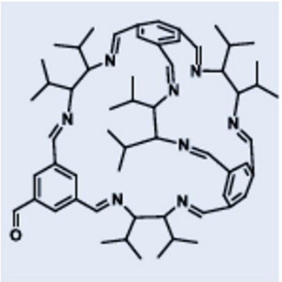

[3+4]

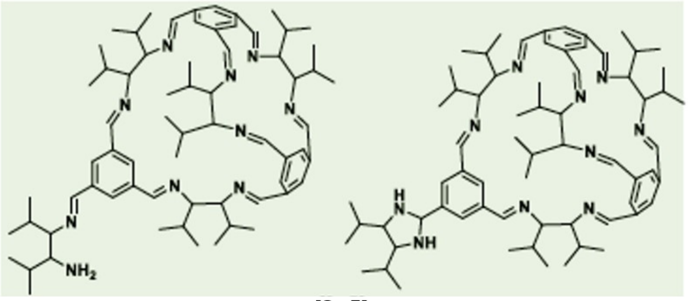

[3+5]

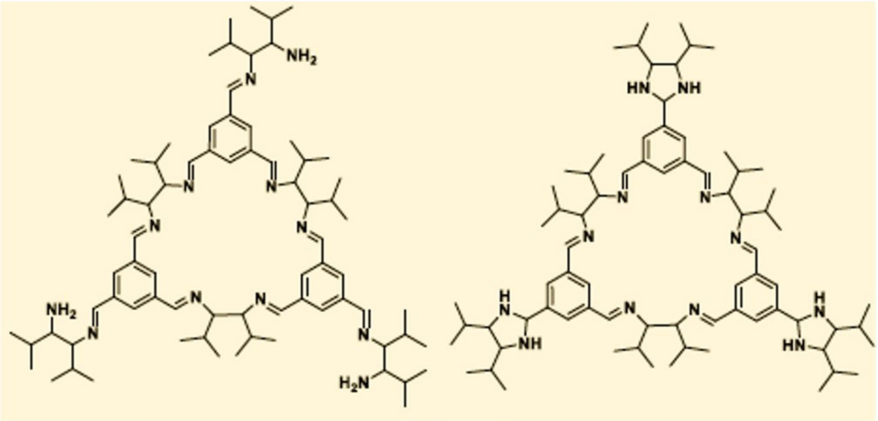

[3+6]

**Figure S7**: Potential structures for the identified intermediates in the HRMS spectra, showing both imine and aminal configurations.

# 3. Control reactions

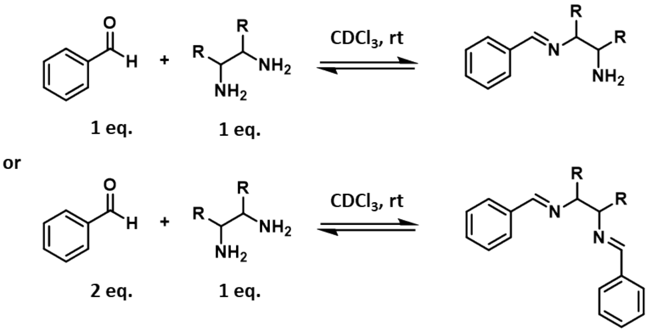


**General procedure:** Benzaldehyde (1 or 2 eq.) was dissolved in $CDCl_3$ (3 mL) and diamine (1 eq.) in $CDCl_3$ (3 mL) was added. The reaction mixture was set to stir at room temperature for 72 hours, before being analysed by $^1$H NMR spectroscopy to determine the composition.

| **Diamine** | Diamine (eq.) | Diamine quantity (mmol) | Diamine quantity (mg) | Benzaldehyde (eq.) | Benzaldehyde quantity (mmol) | Benzaldehyde quantity (mg) |
|---|---|---|---|---|---|---|
| 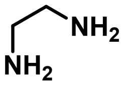 | 1 | 0.205 | 12.4 | 1 | 0.205 | 21.8 |
| | | | | 2 | 0.411 | 43.6 |
| 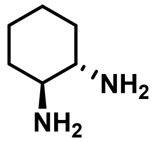 | 1 | 0.205 | 23.5 | 1 | 0.205 | 21.8 |
| | | | | 2 | 0.411 | 43.6 |
| 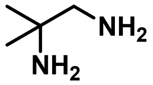 | 1 | 0.205 | 18.1 | 1 | 0.205 | 21.8 |
| | | | | 2 | 0.411 | 43.6 |
| 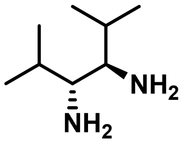 | 1 | 0.205 | 44.6* | 1 | 0.205 | 21.8 |
| | | | | 2 | 0.411 | 43.6 |

*Dihydrochloride salt of diamine used, so triethylamine (0.95 mL, 0.678, 3.3 eq.) added to reaction mixture.

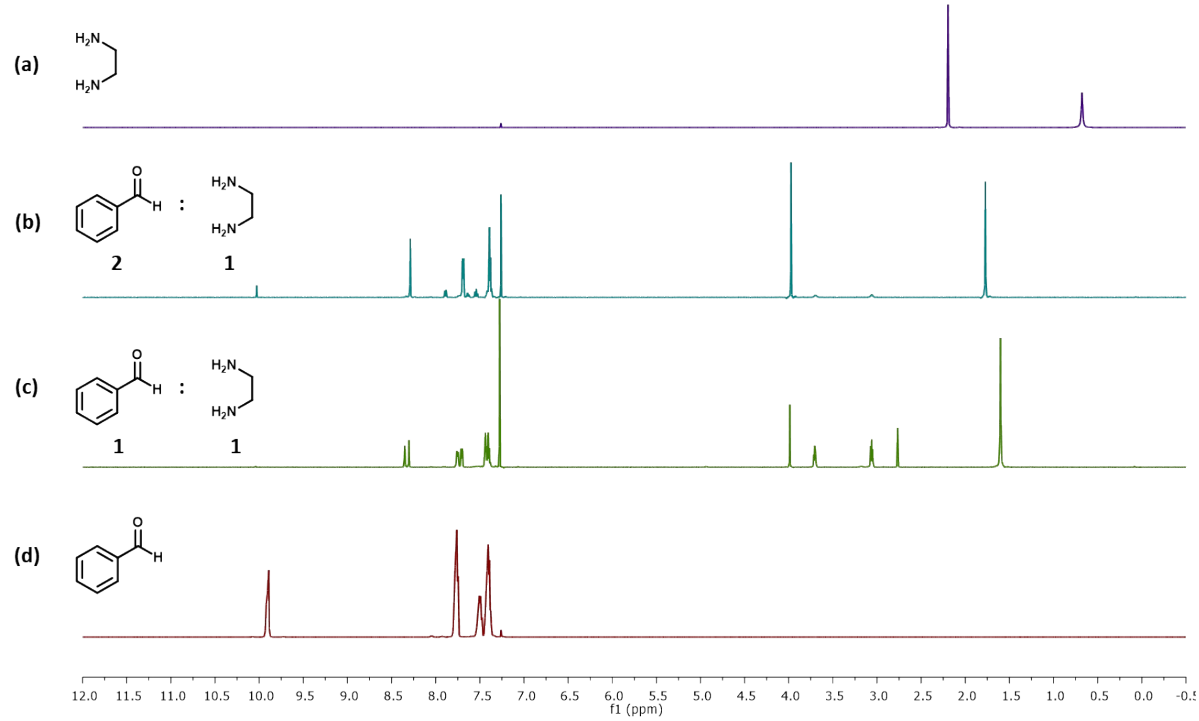


**Figure S8:** Stacked $^{1}H$ NMR spectra ($CDCl_3$) showing the reaction between (a) ethylenediamine (EDA, 1 eq., top) and (d) benzaldehyde (1 or 2 equiv, bottom), with the formation of di-imine (b) or a mixture of mono- and di-imine (c) apparent.

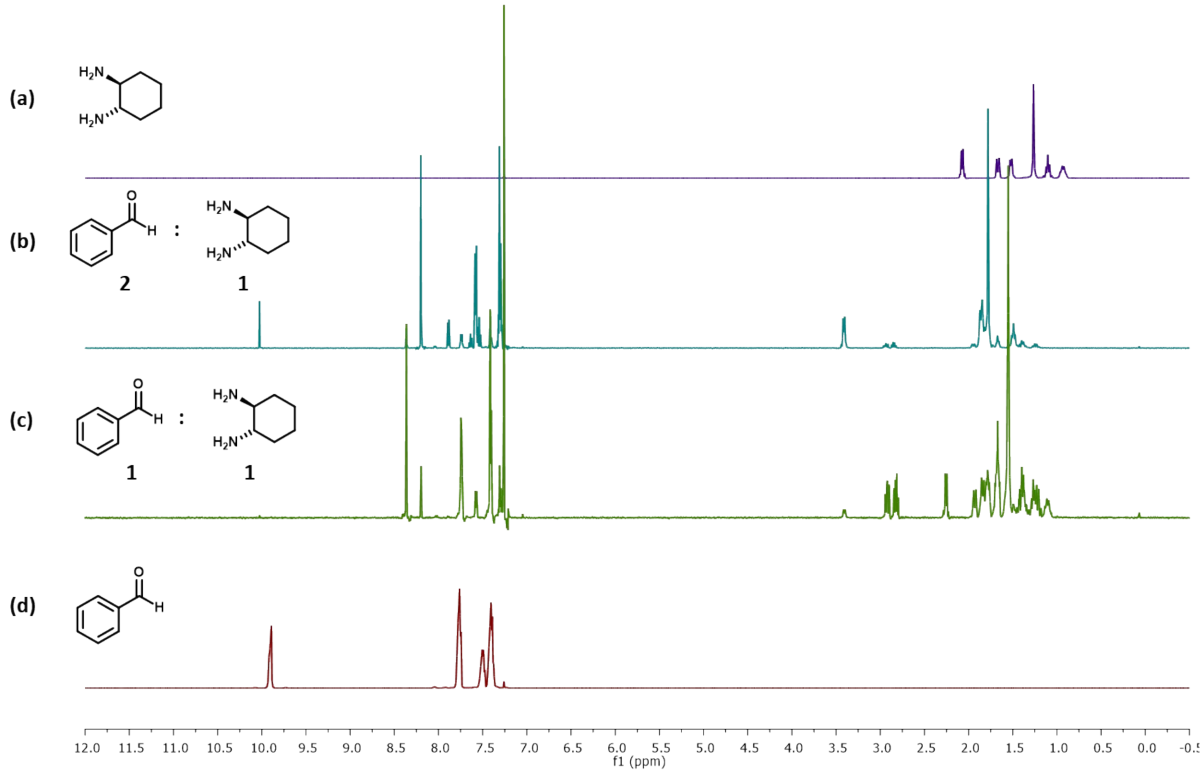


**Figure S9:** Stacked $^{1}H$ NMR spectra ($CDCl_3$) showing the reaction between (a) (1*S*,2*S*)-1,2-cyclohexanediamine (CHDA, 1 eq., top) and (d) benzaldehyde (1 or 2 eq., bottom), with the formation of di-imine and residual aldehyde (b), or a mixture of mono- and di-imine (c), apparent.

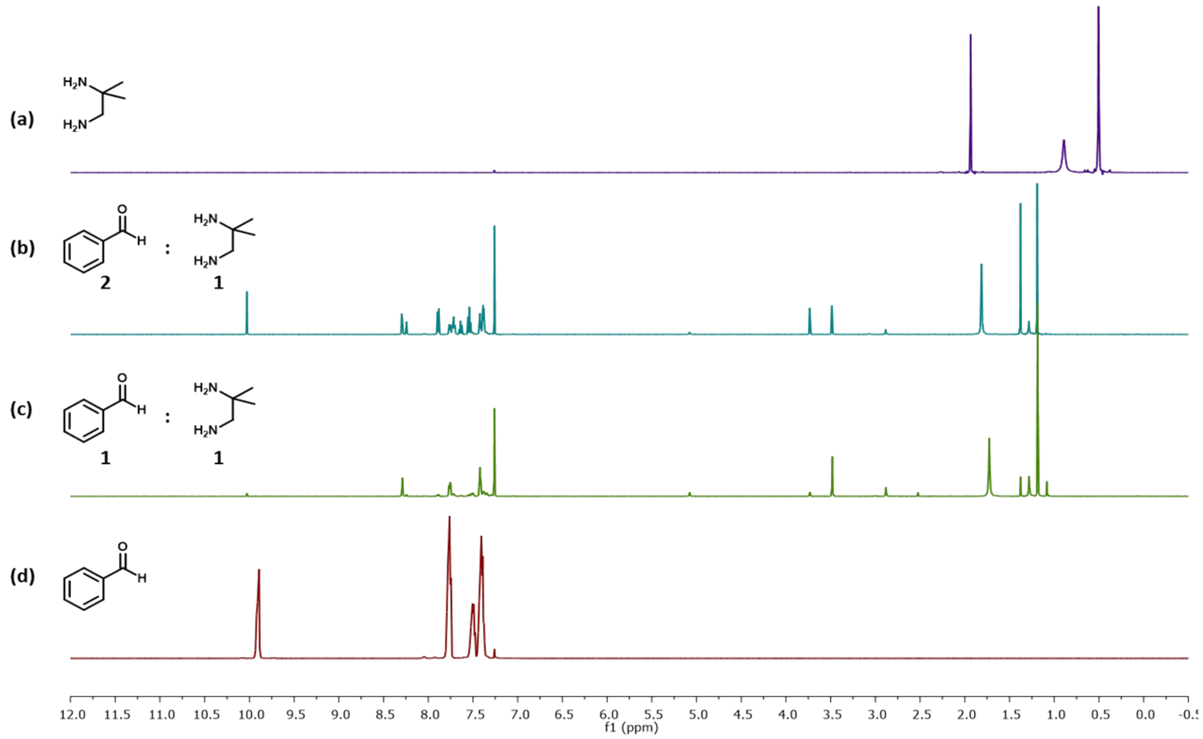


**Figure S10:** Stacked $^1$H NMR spectra ($CDCl_3$) showing the reaction between (a) 2-methylpropane-1,2-diamine (MDPA, 1 eq., top) and (d) benzaldehyde (1 or 2 eq., bottom), with the formation of a mixture of mono- and di-imine and residual aldehyde (b), or mono-imine (c), apparent.

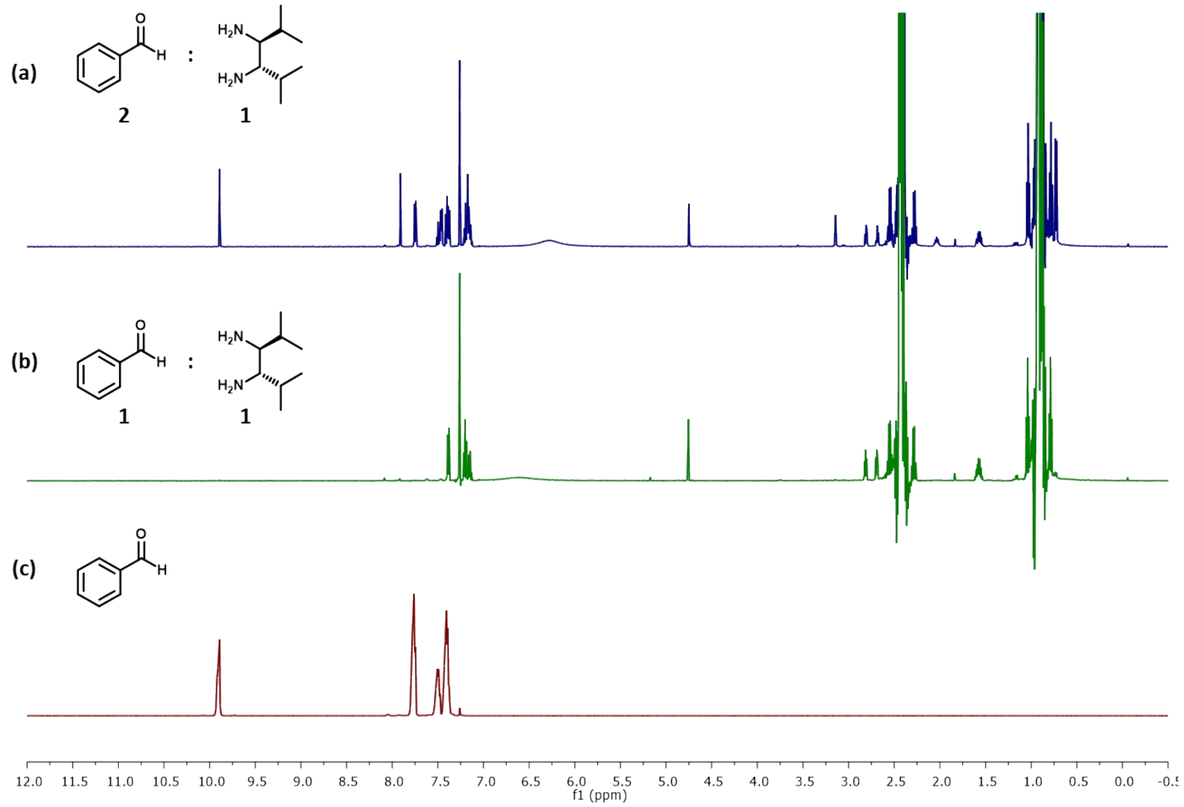


**Figure S11:** Stacked $^1$H NMR spectra ($CDCl_3$) showing the reaction between 2,5-dimethylhexane-3,4-diamine (DMHDA, 1 eq.) and (c) benzaldehyde (1 or 2 eq., bottom), with the formation of a mixture of aminal, imine species, and residual aldehyde (a), or clean conversion to the aminal (b) apparent.

## 4. Precursor Syntheses, Optimisation and Characterisation of CC21

**2,2'-((1*E*,1'*E*)-(((3*R*,4*R*)-2,5-Dimethylhexane-3,4-diyl)bis(azaneylylidene))bis(methaneylylidene))di-phenol:**

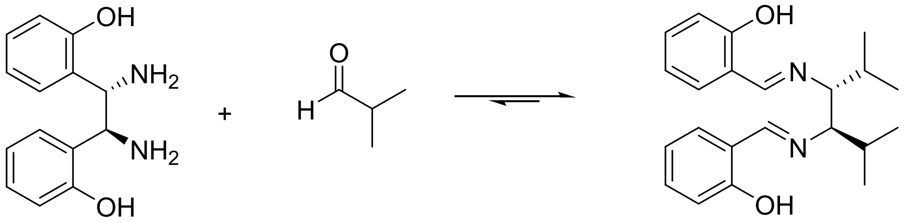


A solution of 1,2-bis-(2-hydroxyphenyl)-1,2-diaminoethane ((*S,S*)-hpen, 10.00 g, 40.9 mmol, 1.0 eq.) and isobutyraldehyde (7.38 g, 102.3 mmol, 2.5 eq.) in toluene (135 mL) was refluxed at 120 °C for 72 hours fitted with a pre-filled Dean-Stark trap. The resulting mixture was allowed to cool to room temperature and the solvent removed under reduced pressure. Methanol was then added to the crude mixture and the product was collected by filtration as a yellow powder (12.20 g, 34.6 mmol, 85%).

**$^1$H NMR** (400 MHz, $CDCl_3$) $\delta_H$ 13.54 (2H, br s), 8.16 (2H, s), 7.27–7.21 (2H, m), 7.13 (2H, dd, *J* = 7.7, 1.6 Hz), 6.93 (2H, d, *J* = 8.0 Hz), 6.78 (2H, t, *J* = 8.0 Hz), 3.22 (2H, s), 2.17–2.03 (2H, m), 0.97 (6H, d, *J* = 6.8 Hz), 0.89 (6H, d, *J* = 6.8 Hz); **$^{13}$C NMR** (101 MHz, $CDCl_3$) $\delta_C$ 165.43, 161.13, 132.02, 131.23, 118.25, 118.22, 116.78, 75.98, 28.23, 20.32, 17.16; **HRMS** (CI+) calculated for $C_{22}H_{28}N_2O_2$ 352.2151, found $[M+H]^+$ 353.2235. Data in agreement with literature values.[4]

**(*3R,4R*)-2,5-Dimethylhexane-3,4-diamine dihydrochloride:**

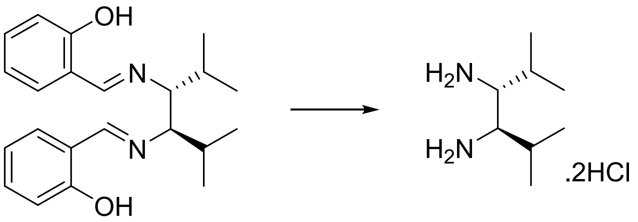


2,2'-((1*E*,1'*E*)-(((3*R*,4*R*)-2,5-Dimethylhexane-3,4-diyl)bis(azaneylylidene))bis(methaneylylidene))di-phenol (3.93 g, 10.3 mmol) was dissolved in THF (50 mL), before a solution of HCl (3 mL, 37%) in THF (10 mL) was added. The reaction mixture was stirred at room temperature for 48 hours and the resulting precipitated product, (*3R,4R*)-2,5-dimethylhexane-3,4-diamine dihydrochloride, was collected by vacuum filtration as a white solid. (1.77 g, 8.2 mmol, 79%).

**$^1$H NMR** (400 MHz, $D_2O$) $\delta_H$ 3.48 (2H, d, *J* = 8.0 Hz), 2.19 (2H, m), 1.10 (12H, t, *J* = 6.6 Hz); **$^{13}$C NMR** (101 MHz, $D_2O$) $\delta_C$ 56.67, 27.26, 18.55, 17.13; **HRMS** (CI+) calculated for $C_8H_{20}N_2$ 144.1626, found $[M+H]^+$ 145.1660. Data in agreement with literature values.[4]

*N.B.* The opposite enantiomer, (*3S,4S*)-2,5-dimethylhexane-3,4-diamine dihydrochloride, can be formed using the same method by starting with (*R*,*R*)-hpen instead.

**1,3,5-Tris((4*R*,5*R*)-4,5-diisopropylimidazolidin-2-yl)benzene:**

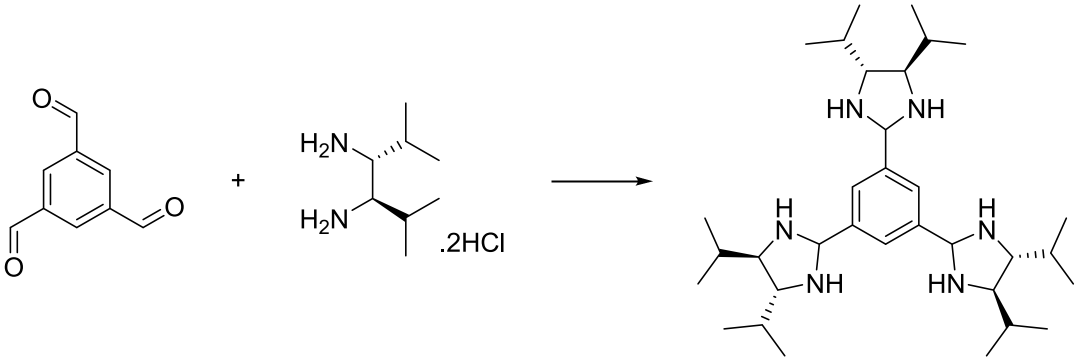


1,3,5-Triformylbenzene (0.5 g, 6.17 mmol, 4 eq.) was dissolved in chloroform (60 mL). (3*R*,4*R*)-2,5-Dimethylhexane-3,4-diamine dihydrochloride (2.01 g, 9.26 mmol, 6 eq.) and triethylamine (0.37 mL, 3.3 eq.) in chloroform (60 mL) was added. The reaction was stirred at room temperature for 72 hours before the solvent was removed *in vacuo*. The purified product was precipitated with methanol and collected by filtration to afford the product as a pink powder (1.11g, 2.05 mmol, 33%).

**$^1$H NMR** (400 MHz, $CDCl_3$) $\delta_H$ 7.59 (3H, s), 4.87 (3H, s), 2.87 (6H, dt, *J* = 40.0, 5.7 Hz), 2.02 (6H, br s), 1.72-1.66 (6H, m), 1.00-0.96 (36H, m); **$^{13}$C NMR** (101 MHz, $CDCl_3$) $\delta_C$ 142.28, 124.88, 67.96, 67.57, 32.75, 32.29, 20.98, 20.86, 18.78, 18.64; **HRMS** (ES+) calculated for $C_{33}H_{60}N_6$ 540.4879, found $[M+H]^+$ 541.4958.

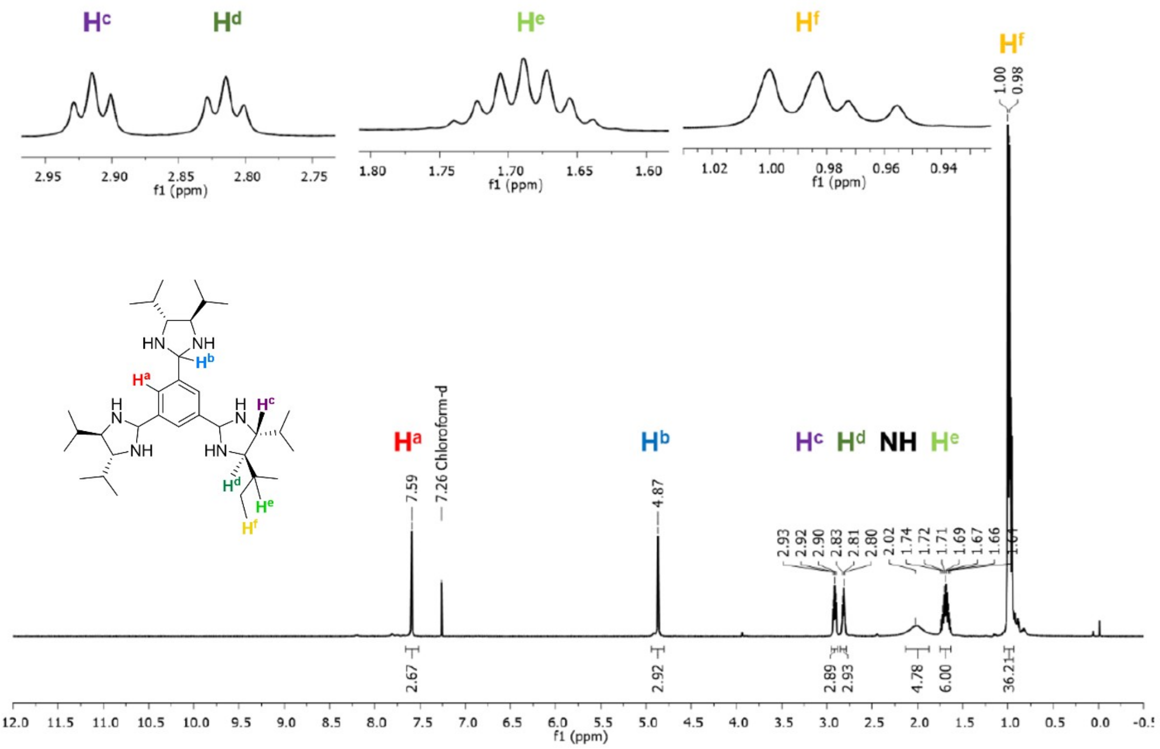

**Figure S12:** $^{1}H$ NMR ($CDCl_3$) of 1,3,5-tris((4*R*,5*R*)-4,5-diisopropylimidazolidin-2-yl)benzene

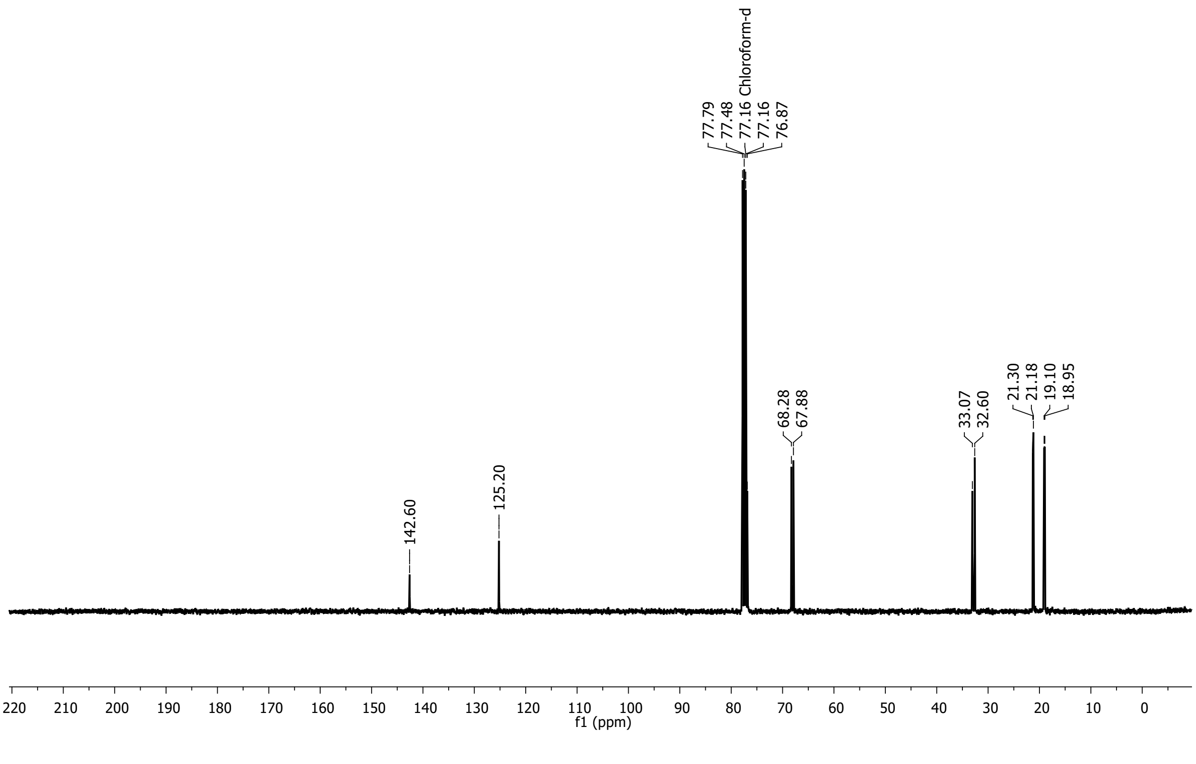

**Figure S13:** $^{13}C$ NMR ($CDCl_3$) spectrum of 1,3,5-tris((4*R*,5*R*)-4,5-diisopropylimidazolidin-2-yl)benzene

**(1*E*,1'*E*,1''*E*)-1,1',1''-(Benzene-1,3,5-triyl)tris(*N*-isobutyl methanimine):**

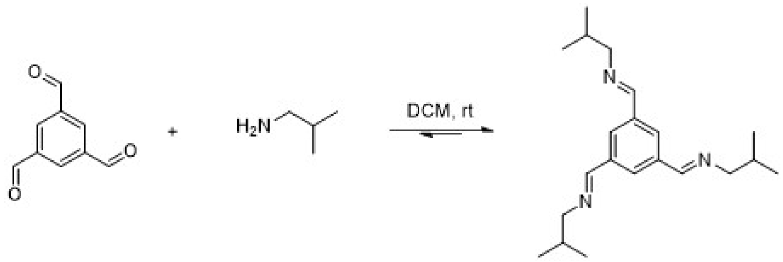


A solution of 1,3,5-triformylbenzene (1.00 g, 6.16 mmol, 1.0 eq.) and isobutylamine (1.9 mL, 19.12 mmol, 3.1 eq.) in DCM (100 mL) was stirred at room temperature for 23 hours. The colourless solution was dried ($K_2CO_3$), filtered, and concentrated *in vacuo* to afford the desired product as a pale yellow oil (1.56 g, 4.78 mmol, 77%) which was used without further purification.

**$^1$H NMR** (400 MHz, $CDCl_3$) $\delta_H$ 8.30 (3H, s), 8.14 (3H, s), 3.45 (6H, dd, *J* = 6.6, 1.3 Hz), 2.02 (3H, dp, *J* = 13.3, 6.7 Hz), 0.95 (18H, d, *J* = 6.7 Hz); **HRMS** (ES+) calculated for $C_{20}H_{31}N_3$ 327.2674, found $[M+H]^+$ 328.3114, $[M+Na]^+$ 350.2466.

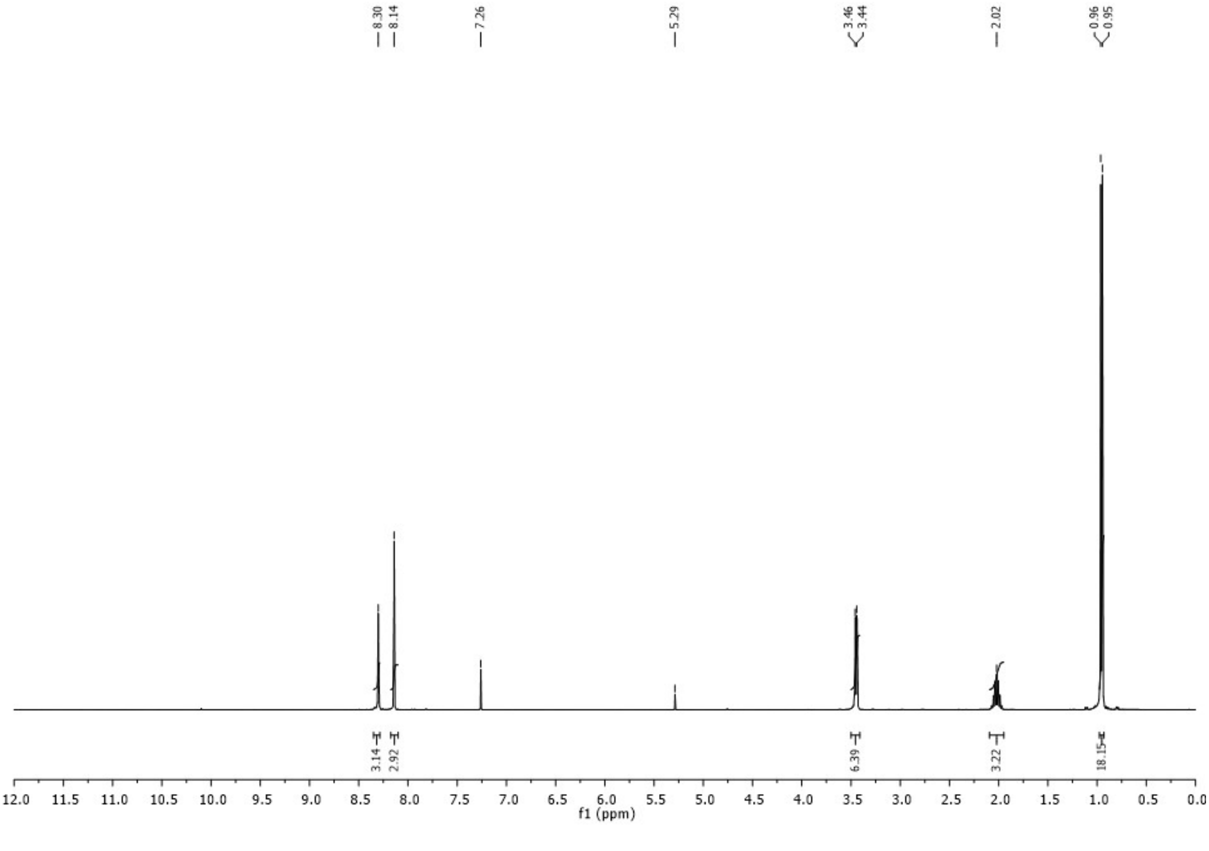


**Figure S14:** $^1$H NMR ($CDCl_3$) of (1*E*,1'*E*,1''*E*)-1,1',1''-(benzene-1,3,5-triyl)tris(*N*-isobutylmethanimine)

**Optimisation Screen:**

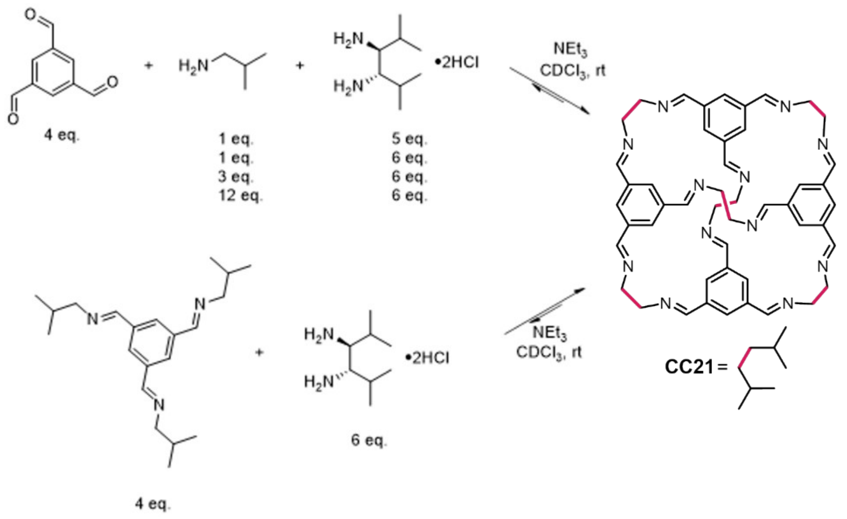


**General methods for optimisation screen:**

**Method 1 – direct formation:** To a solution of 1,3,5-triformylbenzene (0.18 g, 1.11 mmol, 4.0 eq.) in D-chloroform (23 mL), was added solutions of isobutylamine (27-329 µL, 0.28-3.32 mmol, 1.0-12.0 eq.) in D-chloroform (5 mL), and (3*S*,4*S*)-2,5-dimethylhexane-3,4-diamine dihydrochloride (0.30-0.36 g, 1.38-1.66 mmol, 5.0-6.0 eq.) and triethylamine (0.58-0.69 mL, 4.14-4.97 mmol, 15.0-18.0 eq.) in D-chloroform (5 mL). The reaction mixtures were stirred for 2 weeks at room temperature and monitored by $^{1}$H NMR spectroscopy and HRMS.

**Method 2 – formal transimination:** To a solution of (1*E*,1'*E*,1''*E*)-1,1',1''-(benzene-1,3,5-triyl)tris(*N*-isobutylmethanimine) (0.36 g, 1.11 mmol, 4.0 eq.) in D-chloroform (23 mL), was added a solution of (3*S*,4*S*)-2,5-dimethylhexane-3,4-diamine dihydrochloride (0.36 g, 1.66 mmol, 6.0 eq.) and triethylamine (0.69 mL, 4.97 mmol, 18.0 eq.) in D-chloroform (10 mL). The reaction mixtures was stirred for 2 weeks at room temperature and monitored by $^{1}$H NMR spectroscopy and HRMS.

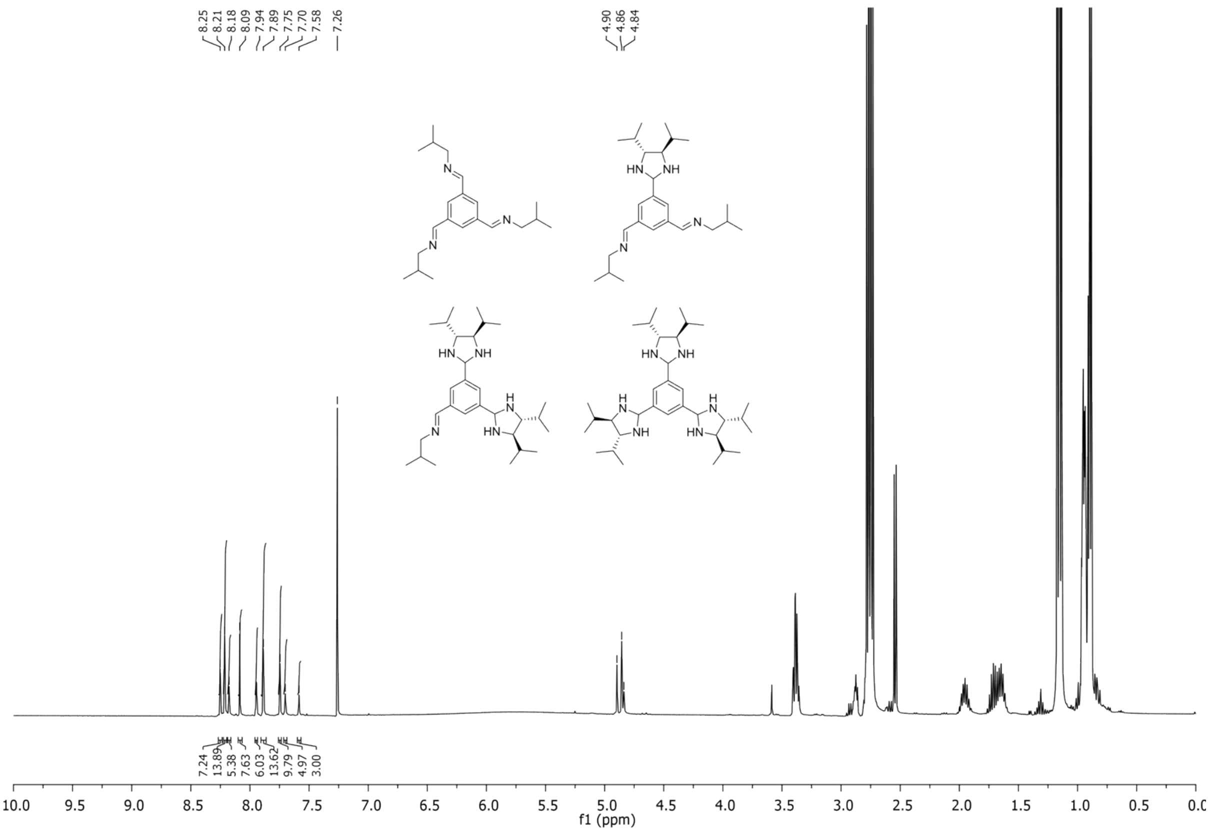


**Figure S15:** $^{1}$H NMR ($CDCl_3$) of the mixture of species formed using the transimination precursor 1*E*,1'*E*,1''*E*)-1,1',1''-(benzene-1,3,5-triyl)tris(*N*-isobutylmethanimine) in the presence of DMHDA – this scrambled distribution formed within 2 days and did not equilibrate further.

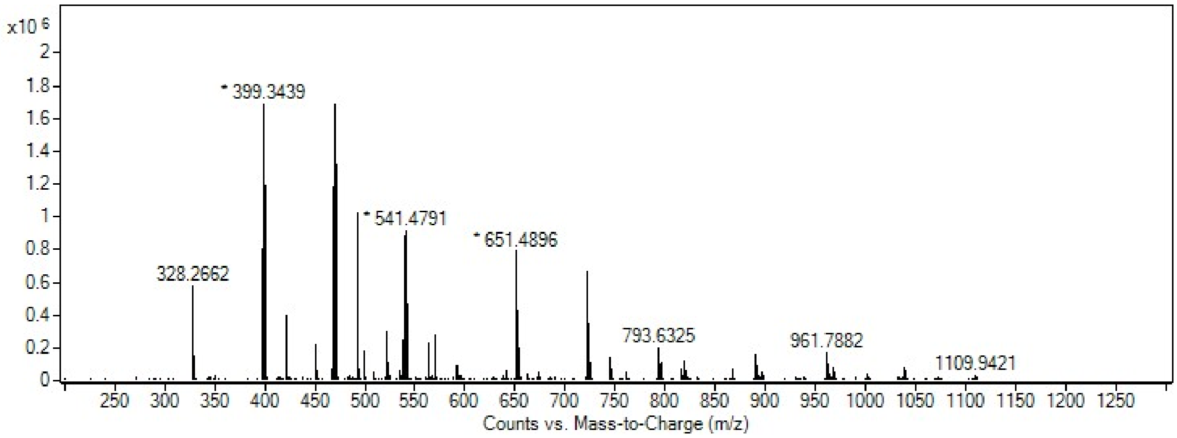


**Figure S16:** HRMS spectra of the mixture of scrambled [1+3] species formed using the transimination precursor 1*E*,1'*E*,1''*E*)-1,1',1''-(benzene-1,3,5-triyl)tris(*N*-isobutylmethanimine) in the presence of DMHDA – mass ions corresponding to the transamination precursor – $[M+H]^+$ 328.2662, the precursor with one isobutylamine linker exchanged for DMHDA – $[M+H]^+$ 399.3439, the precursor with two isobutylamine linkers exchanged for DMHDA – $[M+H]^+$ 470.4146, and 1,3,5-tris((4*S*,5*S*)-4,5-diisopropylimidazolidin-2-yl)benzene – $[M+H]^+$ 541.4791 found.

**Initial Synthesis of CC21** (in the presence of 2-methylpropane-1,2-diamine):

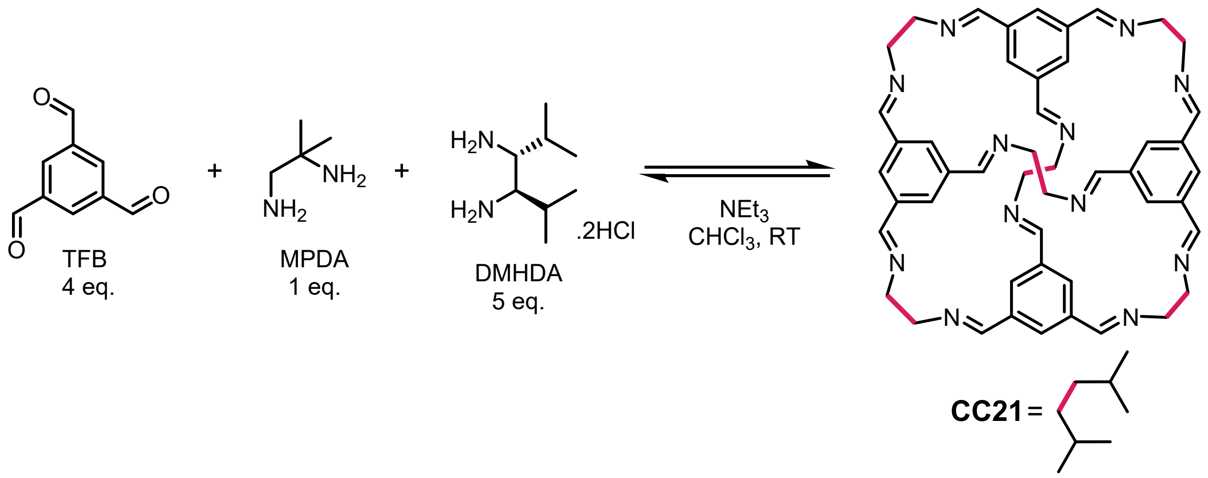


To a solution of 1,3,5-triformylbenzene (0.33 g, 2.055 mmol, 4.0 eq.) in chloroform (30 mL), was added solutions of 2-methylpropane-1,2-diamine (0.045 g, 0.514 mmol, 1.0 eq.) in chloroform (15 mL), and (3*R*,4*R*)-2,5-dimethylhexane-3,4-diamine dihydrochloride (0.59 g, 2.570 mmol, 5 eq.) and triethylamine (0.2 mL, 1.7 mmol, 3.3 eq.) in chloroform (15 mL). The reaction mixture was stirred for 72 hours at room temperature before the solvent was removed under reduced pressure. The crude product was dissolved in THF, filtered to remove triethylamine-hydrochloride salts, and the filtrate concentrated under reduced pressure. The resulting oil was triturated in methanol and the purified parent cage **CC21** was collected as a colourless solid (52 mg, 0.04 mmol, 5%).

**Optimised Synthesis of CC21** (in the presence of isobutylamine):

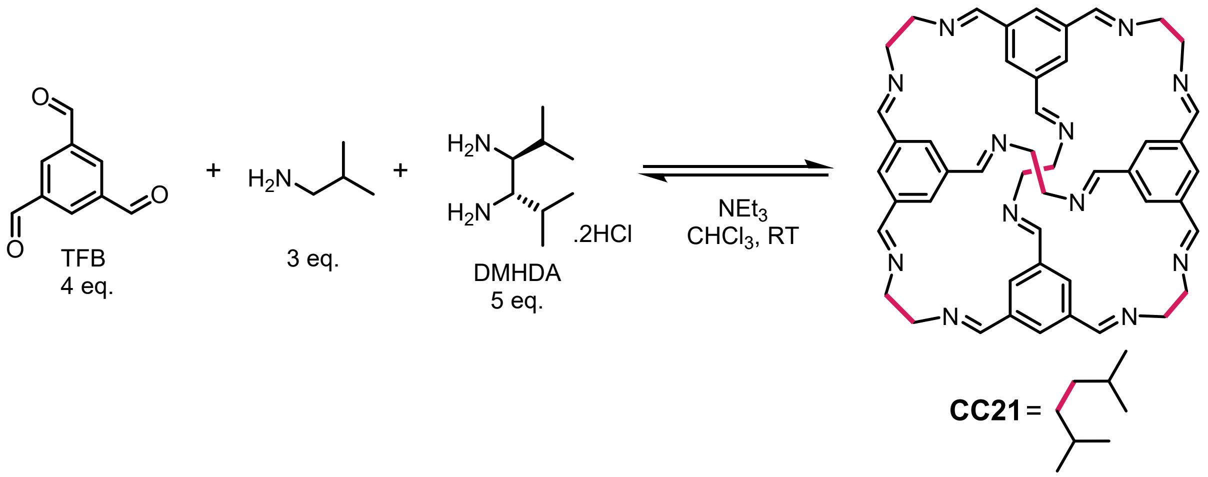


To a solution of 1,3,5-triformylbenzene (0.18 g, 1.11 mmol, 4.0 eq.) in D-chloroform (23 mL), was added solutions of isobutylamine in D-chloroform (5 mL), and (3*S*,4*S*)-2,5-dimethylhexane-3,4-diamine dihydrochloride and triethylamine in D-chloroform (5 mL). The reaction mixture was stirred for 14 days at room temperature before the solvent was removed under reduced pressure. The crude product was dissolved in THF, filtered to remove triethylamine-hydrochloride salts, and the filtrate concentrated under reduced pressure. The resulting material was triturated in methanol and the purified parent cage **CC21** collected as a cream solid (105 mg, 29%).

**IR** ($\nu_{max}$ /cm$^{-1}$): 2959, 2865, 1647, 1595, 1457, 1377, 1243, 1148, 1057, 979, 878, 688; **$^{1}$H NMR** (400 MHz, $CDCl_3$) $\delta_H$ 8.03 (12H, s), 7.88 (12H, s), 3.36 (12H, s), 2.20 (12H, br s), 1.03 (36H, d, *J* = 6.7 Hz), 0.78 (36H, d, *J* = 6.7 Hz); **$^{13}$C NMR** (101 MHz, $CDCl_3$) $\delta_C$ 159.85, 136.76, 129.77, 28.65, 21.59, 16.02; **HRMS** (ES+) calculated for $C_{84}H_{120}N_{12}$ 1296.9790, found $[M+H]^+$ 1298.0221 and $[M+2H]^{2+}$649.5070.

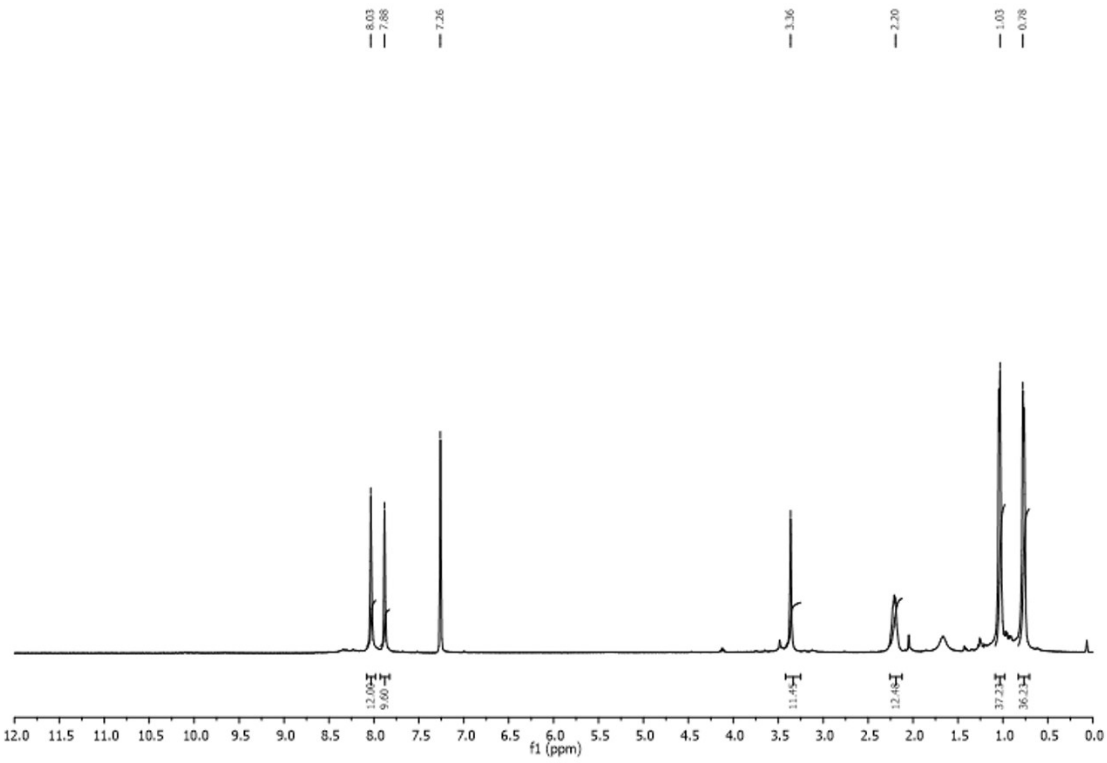


**Figure S17***:* $^{1}H$ NMR ($CDCl_3$) spectrum of **CC21** parent cage.

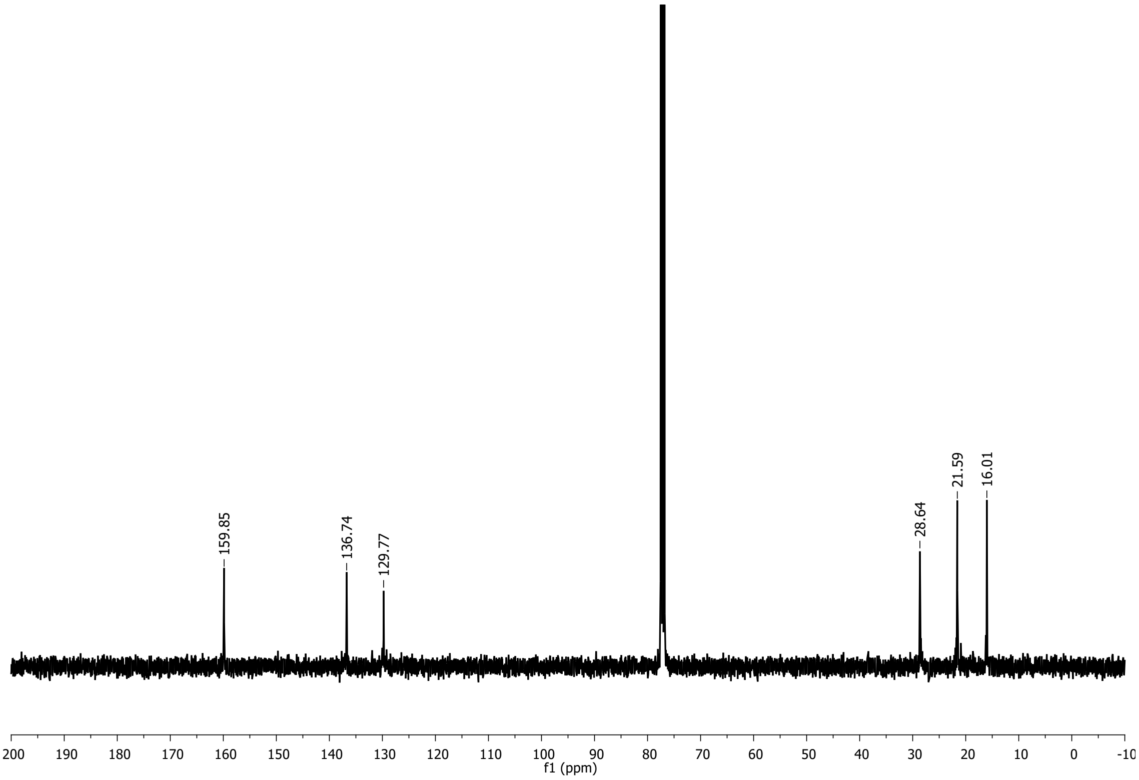


**Figure S18**: $^{13}C$ NMR ($CDCl_3$) spectrum of **CC21** parent cage.

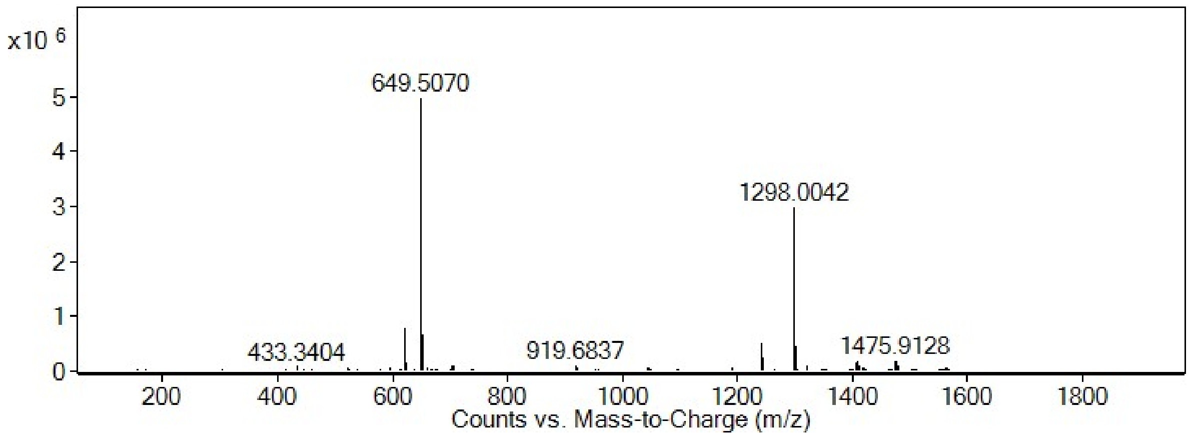


**Figure S19:** HRMS of **CC21** parent cage with the expected mass peaks relating to $[M+H]^+$ at 1298.0042 and $[M+2H]^{2+}$ 649.5070

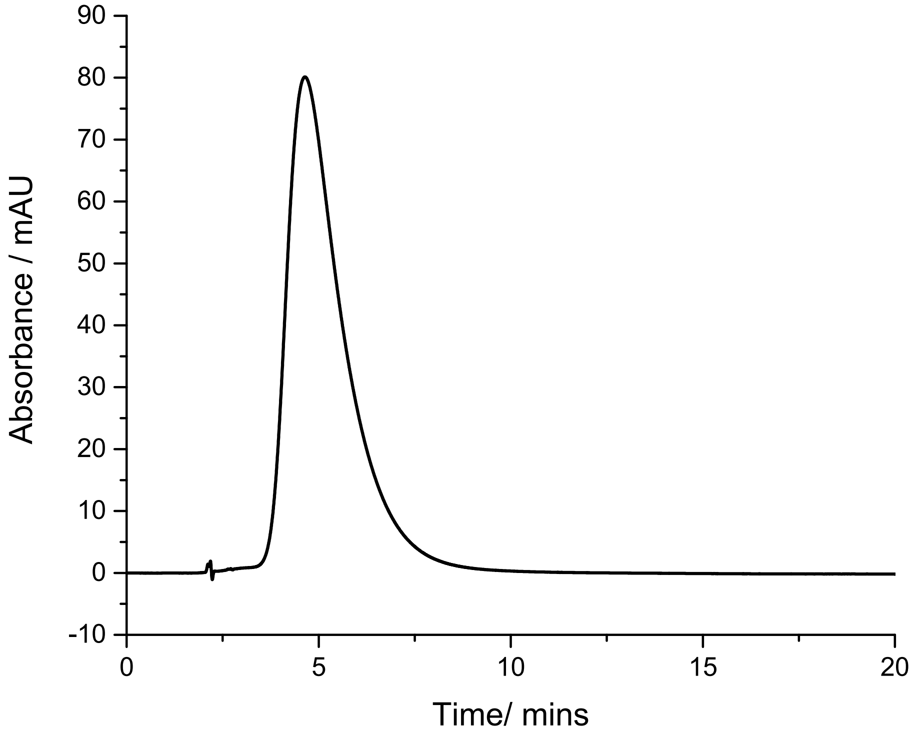


**Figure S20**: HPLC chromatogram of **CC21** parent cage.

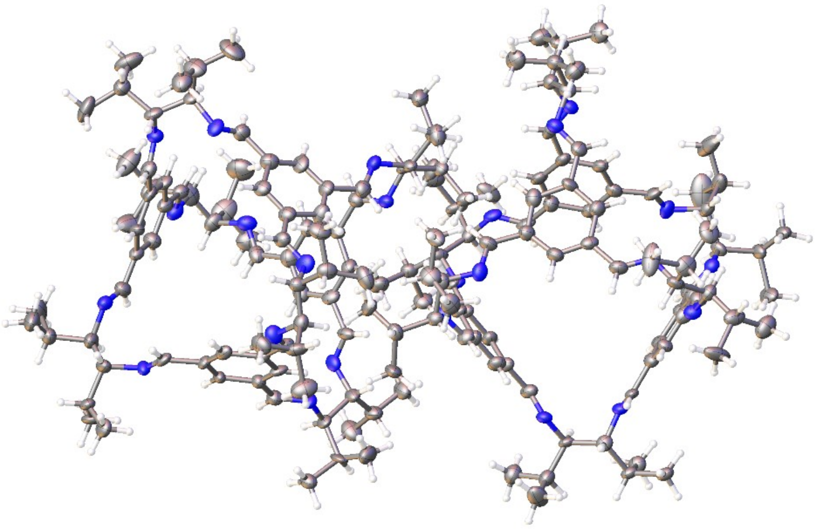

**Figure S21:** Displacement ellipsoid plot of the asymmetric unit from the single crystal structure, 2(**CC21**-*R*)·9($CHCl_3$)·10.5($CH_4O$)·($H_2O$). Ellipsoids displayed at 30% probability level; disordered solvent omitted for clarity. C = grey, H = white, N = blue. All the amine groups are symmetrically equivalent and have the same absolute conformation in the crystal structure. CCDC submission code: 2234276.

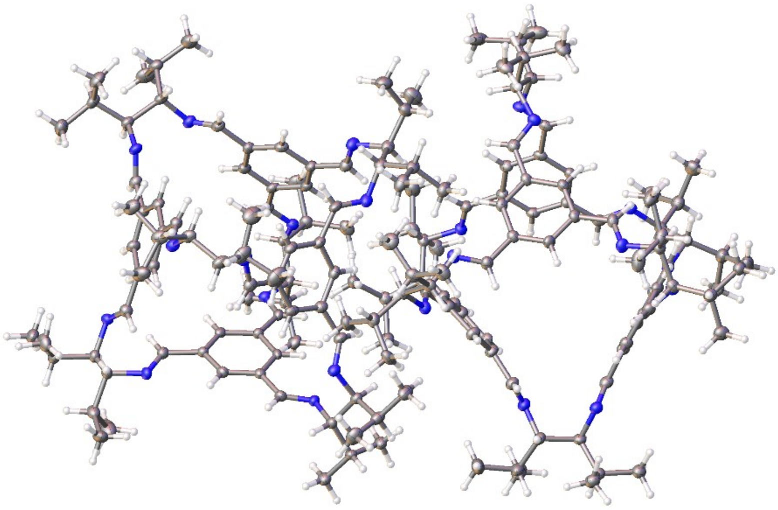

**Figure S22:** Displacement ellipsoid plot of the asymmetric unit from the single crystal structure, **CC21**α from **CC21**-*S*. Ellipsoids displayed at 30% probability level. C = grey, H = white, N = blue. All the amine groups are symmetrically equivalent and have the same absolute conformation in the crystal structure. CCDC entry code: 2234277.

**Table S2**. Single crystal X-ray refinement details for **CC21**·9($CHCl_3$)·10.5($CH_4O$)·($H_2O$) and **CC21**-α.

| | **CC21**·9($CHCl_3$)·10.5($CH_4O$)·($H_2O$) | **CC21**-α[a] |
|---|---|---|
| Crystallisation Solvent | $CHCl_3$/MeOH | |
| Space Group | $P2_1$ | $P2_1$ |
| Wavelength [Å] | Mo-Kα | Mo-Kα |
| Collection Temperature | 100 K | 100 K |
| Formula | 2($C_{84}H_{120}N_{12}$)·9($CHCl_3$)·10.5($CH_4O$)·($H_2O$) | 2(C84 H120 N12) |
| *Mr* | 4024.59 | |
| Crystal Size (mm) | 0.31 x 0.27 x 0.07 | 0.20 x 0.16 x 0.04 |
| Crystal System | Monoclinic | Monoclinic |
| *a* [Å] | 16.0941(13) | 14.7314(3) |
| *b* [Å] | 45.047(4) | 41.6927(8) |
| *c* [Å] | 16.4682(15) | 15.9578(3) |
| *α* [°] | | |
| *β* [°] | 105.336(2) | 94.0143(19) |
| *γ* [°] | | |
| *V* [$Å^3$] | 11514.2(17) | 9777.1(3) |
| *Z* | 2 | 2 |
| $D_{calcd}$ [g $cm^{-3}$] | 1.161 | 0.882 |
| *μ* [$mm^{-1}$] | 0.373 | 0.052 |
| *F*(000) | 4274 | 2832 |
| 2θ range [°] | 2.72 – 46.51 | 3.39 – 46.58 |
| Reflections collected | 84088 | 86289 |
| Independent reflections, $R_{int}$ | 27498, 0.0589 | 27809, 0.1257 |
| Obs. Data [$I > 2\sigma$] | 18041 | 17782 |
| Data / restraints / parameters | 27498 / 154 / 2135 | 27809 / 1 / 1777 |
| Final R1 values ($I > 2\sigma(I)$) | 0.0999 | 0.0660 |
| Final R1 values (all data) | 0.1259 | 0.1257 |
| Final wR($F^2$) values (all data) | 0.2647 | 0.1399 |
| Goodness-of-fit on $F^2$ | 1.500 | 1.005 |
| Largest difference peak and hole [e.$A^{-3}$] | 0.421/-0.353 | 0.354 / -0.166 |
| CCDC | 2234276 | 2234277 |

[a] Recorded after desolvating the crystal of **CC21**—isolated by filtration from $CH_2Cl_2$—at 353 K and then running gas sorption analysis at 77.3 K, crystals of **CC21**-α were weakly diffracting at high angle. Consequently, a resolution limit of 0.9 Å was applied during refinement.

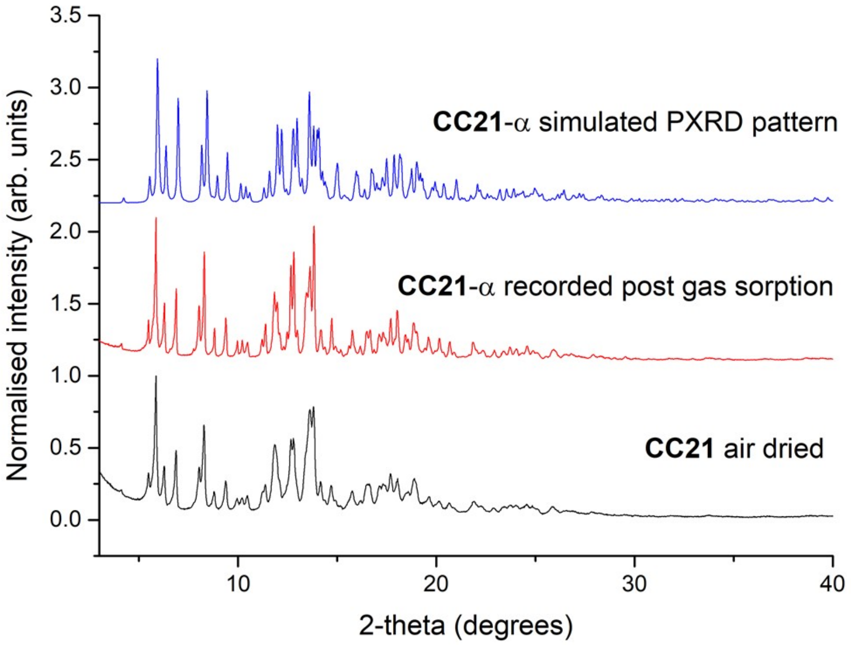


**Figure S23:** PXRD patterns for **CC21**: crystallised from $CH_2Cl_2$ and air dried (bottom, black); recorded after activation at 363 K under dynamic vacuum and subjected to gas sorption analysis (middle, red); simulated pattern for **CC21**α from the single crystal structure recorded at 100 K.

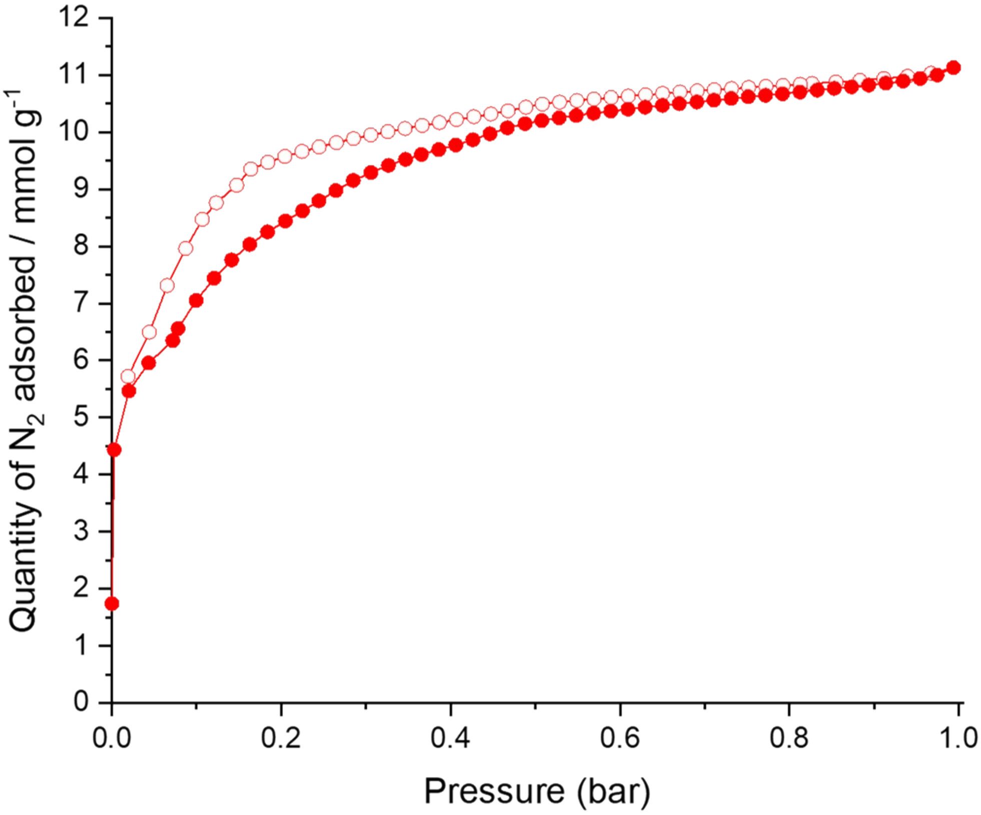


**Figure S24:** $N_2$ sorption isotherm for **CC21**α recorded at 77.3 K. Adsorption points are shown are closed symbols; desorption points are shown as open points.

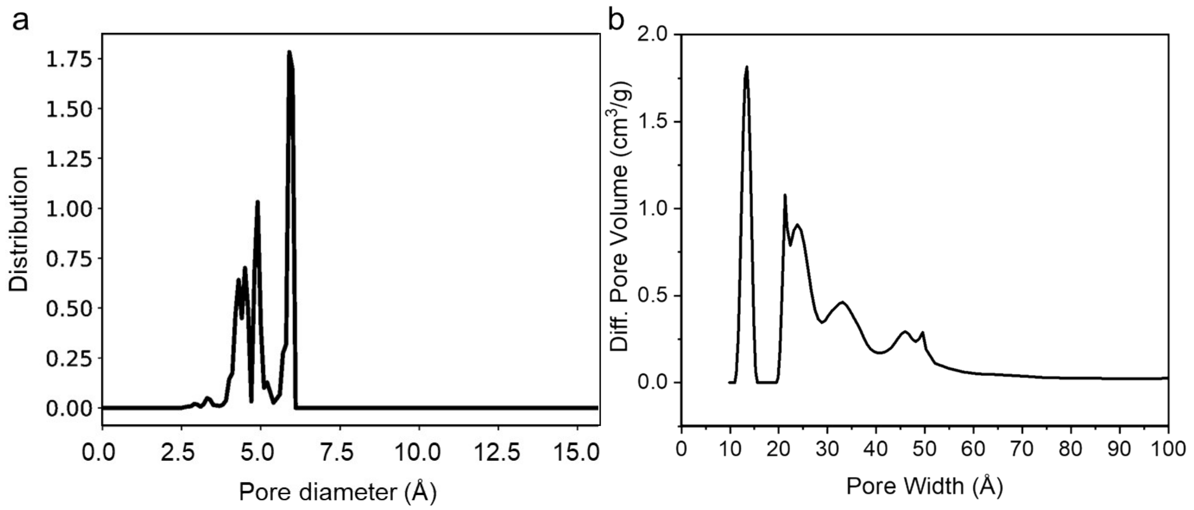


**Figure S25:** a) Calculated PSD of **CC21**α from the crystal structure using a probe radius of 1.0 Å; b) Experimental PSD of **CC21**α recorded at 77.3 K. The calculated PSD assumes a perfect crystal from the crystal structure, where in reality the experimental PSD will be a mixture of defects, interparticle spacing, and cracks that will alter the PSD plot by volume.a

## 5. Computational conformer analysis

To evaluate the preference for aminal formation of a series of diamines, we have performed structural analysis of their conformer ensembles. The script “conformer_analysis.py” in https://github.com/andrewtarzia/intermediate_tester analyses the structural and energetic properties of conformers of the diamines in Table S3. For each diamine, we also analysed the effect of reacting one amine with benzaldehyde, leading to one imine formed per diamine. This mimics the control reactions performed experimentally. For each SMILES string, we used the Python library RDKit[5] to "add hydrogens" (AddHs) and generate 500 conformers using ETKDG version 3[6] with random starting coordinates (“UseRandomCoords” setting). Each conformer is then geometry optimised in the gas phase using the semiempirical density-functional tight-binding method GFN2-xTB (version 6.3.2 was used throughout).[7] The geometry optimisation was performed through our software stko (https://github.com/JelfsMaterialsGroup/stko) with “normal” convergence criteria (corresponding to a change in energy and gradient norm less than 5E-6 $E_h$ and 1E-3 $E_h$bohr$^{-1}$, respectively). For each molecule, we analysed the total free energy and structural properties (N-N distance, N-C-C-N dihedral; Figure S25) of all geometry optimised conformers. Figure S25 shows distributions of all properties for all conformers of all molecules, while we focus on only conformers within 10 kJ mol$^{-1}$ of the lowest energy conformer in Figures 4 and S26.

**Table S3**: SMILES strings of all molecules tested.

| name | diamine SMILES | imine SMILES |
|---|---|---|
| DMHDA | CC(C)[C@H](N)[C@@H](N)C(C)C | CC(C)[C@H](N)[C@@H](/N=C/c1ccccc1)C(C)C |
| MPDA | CC(C)(N)CN | CC(C)(N)C/N=C/c1ccccc1 |
| CHDA | N[C@@H]1CCCC[C@H]1N | N[C@@H]1CCCC[C@H]1/N=C/c1ccccc1 |
| EDA | NCCN | NCC/N=C\\c1ccccc1 |

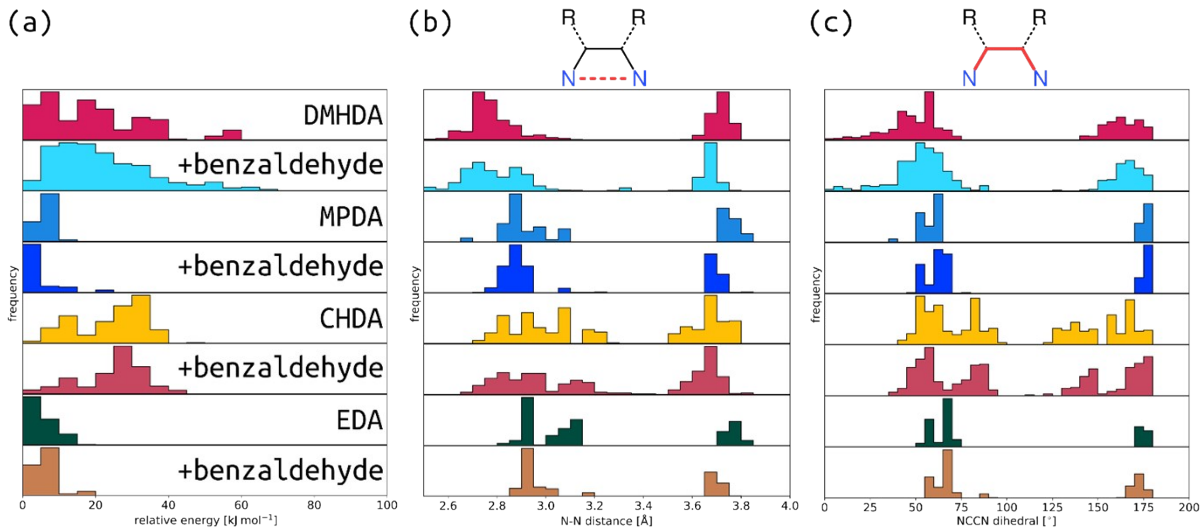


**Figure S26:** (a) Relative free energies, (b) N-N distances and (c) N-C-C-N dihedral angles of all conformers of all molecules in Table S2. Structural properties are shown schematically at the top. Figures 4 and S26 are equivalent to (b) and (c) showing only conformers within the lowest 10 kJ mol$^{-1}$ of the global minimum for each molecule and ignoring the “+benzaldehyde” cases.

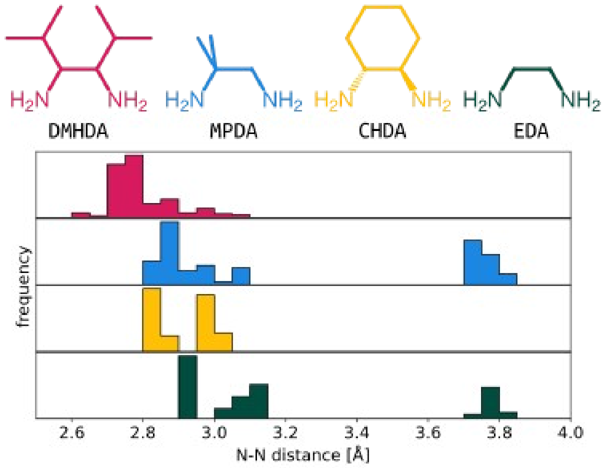


**Figure S27** N-N distances of conformers within the lowest 10 kJ mol$^{-1}$ of the global minimum for each diamine.

**Intermediate landscape analysis**

All intermediate structures (Table S4) were built by modifying cage structures constructed (with *stk*) from TFB and the four amines (DMHDA, MPDA, CHDA and EDA). For all intermediates with a free amine, the aminal version of the intermediate was also constructed. Each intermediate was geometry optimised using the following sequence:

1. The structure was geometry optimised using Schrödinger's MacroModel[8] software and the OPLS3e[9] force field.
2. A conformer search was performed using molecular dynamics (MD) in Schrödinger's MacroModel[8] in the NVE ensemble for 0.5 ns after 10 ps equilibration with a time step of 0.5 fs and a temperature of 700 K. The OPLS3e force field was used. 1000 conformers were extracted from the MD trajectory and optimised.
3. The lowest energy conformer was then geometry optimised using GFN2-xTB[7] (version 6.3.2) to the "extreme" level (corresponding to a change in energy and gradient norm less than 5E-8 $E_h$ and 5E-5 $E_h$bohr$^{-1}$, respectively) in the gas phase.

**Table S4**: Reactions used. [*m*+*n*]-a is the aminal intermediate with *m* tritopic building blocks and *n* ditopic building blocks.

| Intermediate | num. waters/num. imines (*x*) |
|---|---|
| [1+2] | 2 |
| [1+2]-a | 2 |
| [1+3] | 3 |
| [1+3]-a | 3 |
| [2+3] | 5 |
| [2+3]-a | 5 |
| [2+4] | 6 |
| [2+4]-a | 6 |
| [3+4] | 8 |
| [3+5] | 9 |
| [3+5]-a | 9 |
| [3+6] | 9 |
| [3+6]-a | 9 |
| [4+6] | 12 |

Energies were calculated using GFN2-xTB in the gas phase and using the GBSA solvent model (solvent: $CHCl_3$) on GFN2-xTB geometry optimised structures – we used GFN2-xTB due to the technical and cost complexity of applying higher-level of theories to these number and size of structures. All raw energy values and all structures and calculation outputs are available at https://github.com/andrewtarzia/citable_data/tree/master/kearsey_2022. A zenodo DOI is here:

"Andrew Tarzia. (2022). andrewtarzia/citable_data: kearsey_2022_submitted (pocs). Zenodo. https://doi.org/10.5281/zenodo.6790519.

The formation energies of all intermediates were calculated as

$$\text{formation energy} = (E_{\text{cage}} + x(E_{\text{water}})) - (m(E_{\text{BB3}}) + n(E_{\text{BB2}})),$$

where $E_{\text{cage}}$ is the total energy of the cage (or intermediate), $E_{\text{water}}$ is the energy of water, $E_{\text{BB2}}$ is the energy of the ditopic building block, $E_{\text{BB3}}$ is tritopic building block, and $x$, $m$, $n$ are the number of waters produced (imines formed), tritopic building block used, ditopic building block used, respectively.

We found that solvent models did not alter the qualitative energy comparisons of the systems and focused on gas-phase comparisons from here on. The GFN2-xTB gas-phase intermediate formation energies agree, qualitatively, with work by Zhu *et al.* on POC landscapes.[10] The energies obtained from the GFN2-xTB method suggest these cages have similar relative intermediate stabilities. Figure S27 shows the landscapes for all four amines calculated using GFN2-xTB geometry optimised structures in the gas phase. In all cases, the [4+6] cage has the lowest formation energy, when not considering the aminal intermediates.

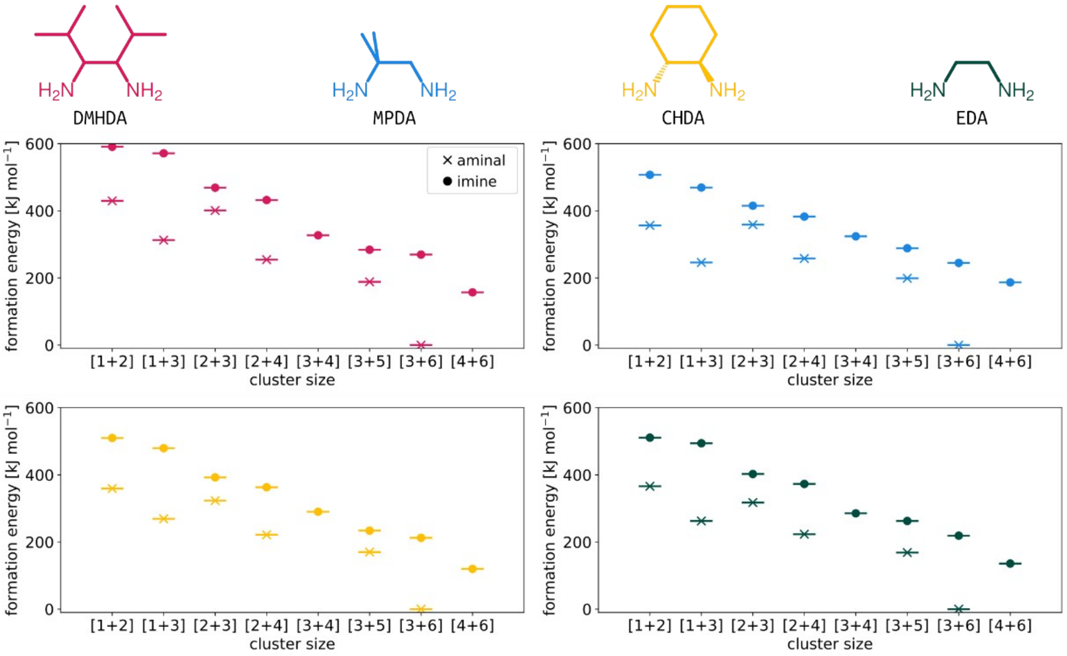


**Figure S28**: Formation energy landscapes of GFN2-xTB geometry optimised structures at the GFN2-xTB level of theory for all four amines. All energies are relative to the minimum energy for that system. Crosses are the aminal species, circles are the imine species.

**Structural porosity analysis**

The desolvated crystal structure **CC21**α was analysed using Zeo++.[11] High accuracy atom radii ("-ha" flag) were used for all calculations. Figure S28(a) shows the accessible and non-accessible surface area of this crystal structure as a function of probe radius. Between a probe radius of 1.55 and 1.6 Å, the pores become non-accessible. The "end-on" radius of $N_2$ is 1.55 Å, while the kinetic radius is 1.82 Å.[12] Based on these radii, the channels of this structure are likely to be open to $N_2$, as seen in the experiments, which will be aided by slight expansion and flexibility in the crystal structure under adsorption conditions. Figure S28(b)—(d) shows the porous network for a series of probe radii. These calculations can be run using the scripts available here https://github.com/andrewtarzia/intermediate_tester/tree/master/porosity_analysis.

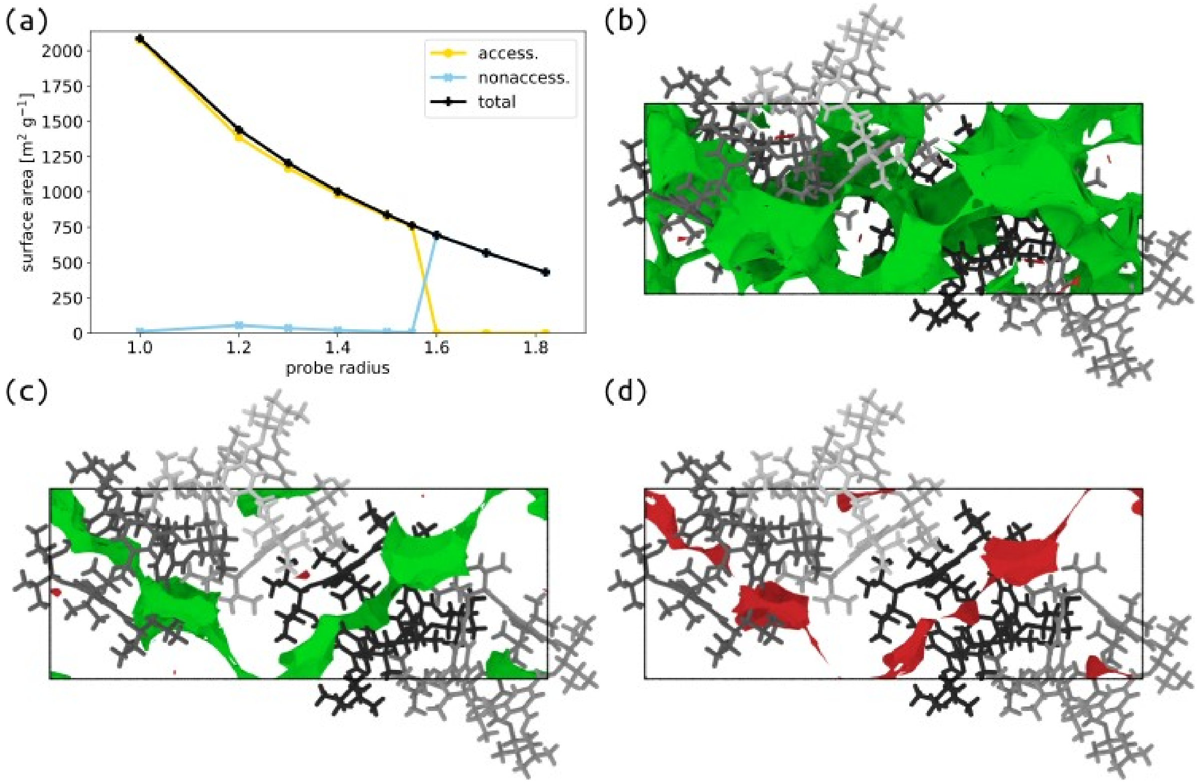


**Figure S29**: (a) Surface area as function of probe radius. Visualisation of accessible (green) and non-accessible (red) surface area in the crystal structure (cages are shown in different grey shades) for a probe radius of (b) 1.0, (c) 1.55 and (d) 1.82 Å. Surfaces were generated with OVITO.[14]

**Automated peak peaking from MS data**

To generate Figure 2 in the manuscript, we implemented automatic peak picking using a Python script (available here: https://github.com/andrewtarzia/intermediate_tester/tree/master/mass_spec_analysis) and the scipy signal library.[13] Given the output from the MS experiment (the ion counts at each mass-to-charge ratio), the script converts the data into relative counts by dividing by the total number of counts in the data set. This conversion allows comparison at different time points and for different experiments. Peaks are extracted using "find_peaks" and a height of 0.001 relative counts. The height of the peaks are not quantified in different experiments, only their presence to show which species are present. We quantify the presence of the species in Table S5 based on the approximate mass values in that table.

**Table S5**: Species and their approximate mass values used in peak picking.

| Species | Approximate Mass Ions |
|---|---|
| [1+2] | 415 |
| [1+3] | 540 |
| [2+3] | 666 |
| [2+4] | 793 |
| [3+4] | 919 |
| [3+5] | 1045 |
| [3+6] | 1189 |
| [4+6] | 1297 |

## References


1 G. M. Sheldrick, *Acta Crystallogr. Sect. A Found. Crystallogr.*, 2015, **71**, 3–8.

2 G. M. Sheldrick, *Acta Crystallogr. Sect. C Struct. Chem.*, 2015, **71**, 3–8.

3 O. V. Dolomanov, L. J. Bourhis, R. J. Gildea, J. A. K. Howard and H. Puschmann, *J. Appl. Crystallogr.*, 2009, **42**, 339–341.

4 R. J. Kearsey, B. M. Alston, M. E. Briggs, R. L. Greenaway and A. I. Cooper, *Chem. Sci.*, 2019, **10**, 9454–9465.

5 G. A. Landrum, RDKit: Open-Source Cheminformatics, http://www.rdkit.org/, (accessed March 1, 2020).

6 S. Wang, J. Witek, G. A. Landrum and S. Riniker, *J. Chem. Inf. Model.*, 2020, **60**, 2044–2058.

7 C. Bannwarth, S. Ehlert and S. Grimme, *J. Chem. Theory Comput.*, 2019, **15**, 1652–1671.

8 Schrödinger Release 2018-4: MacroModel, Schrödinger LLC, New York, NY, 2020

9 K. Roos, C. Wu, W. Damm, M. Reboul, J. M. Stevenson, C. Lu, M. K. Dahlgren, S. Mondal, W. Chen, L. Wang, R. Abel, R. A. Friesner and E. D. Harder, *J. Chem. Theory Comput.*, 2019, **15**, 1863–1874.

10 G. Zhu, Y. Liu, L. Flores, Z. R. Lee, C. W. Jones, D. A. Dixon, D. S. Sholl and R. P. Lively, *Chem. Mater.*, 2018, **30**, 262–272.

11 T. F. Willems, C. H. Rycroft, M. Kazi, J. C. Meza and M. Haranczyk, *Microporous Mesoporous Mater.*, 2012, **149**, 134–141.

12 L. M. Robeson, *J. Memb. Sci.*, 1991, **62**, 165–185.

13 P. Virtanen, R. Gommers, T. E. Oliphant, M. Haberland, T. Reddy, D. Cournapeau, E. Burovski, P. Peterson, W. Weckesser, J. Bright, S. J. van der Walt, M. Brett, J. Wilson, K. J. Millman, N. Mayorov, A. R. J. Nelson, E. Jones, R. Kern, E. Larson, C. J. Carey, İ. Polat, Y. Feng, E. W. Moore, J. VanderPlas, D. Laxalde, J. Perktold, R. Cimrman, I. Henriksen, E. A. Quintero, C. R. Harris, A. M. Archibald, A. H. Ribeiro, F. Pedregosa, P. van Mulbregt, A. Vijaykumar, A. Pietro Bardelli, A. Rothberg, A. Hilboll, A. Kloeckner, A. Scopatz, A. Lee, A. Rokem, C. N. Woods, C. Fulton, C. Masson, C. Häggström, C. Fitzgerald, D. A. Nicholson, D. R. Hagen, D. V. Pasechnik, E. Olivetti, E. Martin, E. Wieser, F. Silva, F. Lenders, F. Wilhelm, G. Young, G. A. Price, G.-L. Ingold, G. E. Allen, G. R. Lee, H. Audren, I. Probst, J. P. Dietrich, J. Silterra, J. T. Webber, J. Slavič, J. Nothman, J. Buchner, J. Kulick, J. L. Schönberger, J. V. de Miranda Cardoso, J. Reimer, J. Harrington, J. L. C. Rodríguez, J. Nunez-Iglesias, J. Kuczynski, K. Tritz, M. Thoma, M. Newville, M. Kümmerer, M. Bolingbroke, M. Tartre, M. Pak, N. J. Smith, N. Nowaczyk, N. Shebanov, O. Pavlyk, P. A. Brodtkorb, P. Lee, R. T. McGibbon, R. Feldbauer, S. Lewis, S. Tygier, S. Sievert, S. Vigna, S. Peterson, S. More, T. Pudlik, T. Oshima, T. J. Pingel, T. P. Robitaille, T. Spura, T. R. Jones, T. Cera, T. Leslie, T. Zito, T. Krauss, U. Upadhyay, Y. O. Halchenko and Y. Vázquez-Baeza, *Nat. Methods*, 2020, **17**, 261–272.

14 A. Stukowski, *Modelling Simul. Mater. Sci. Eng.*, 2010, **18**, 015012.
